# Human myofibroblasts increase the arrhythmogenic potential of human induced pluripotent stem cell-derived cardiomyocytes

**DOI:** 10.1007/s00018-023-04924-3

**Published:** 2023-09-05

**Authors:** Robert D. Johnson, Ming Lei, John H. McVey, Patrizia Camelliti

**Affiliations:** 1 School of Biosciences, University of Surrey, Guildford, UK; 2 Department of Pharmacology, University of Oxford, Oxford, UK

**Keywords:** Cardiac cell therapy, Myocardial infarction, Heterocellular communication, Connexin-43, Interleukin-6, Fibroblasts, Crosstalk, Paracrine

## Abstract

Human induced pluripotent stem cell-derived cardiomyocytes (hiPSC-CMs) have the potential to remuscularize infarcted hearts but their arrhythmogenicity remains an obstacle to safe transplantation. Myofibroblasts are the predominant cell-type in the infarcted myocardium but their impact on transplanted hiPSC-CMs remains poorly defined. Here, we investigate the effect of myofibroblasts on hiPSC-CMs electrophysiology and Ca^2+^ handling using optical mapping of advanced human cell coculture systems mimicking cell–cell interaction modalities. Human myofibroblasts altered the electrophysiology and Ca^2+^ handling of hiPSC-CMs and downregulated mRNAs encoding voltage channels (K_V_4.3, K_V_11.1 and Kir6.2) and SERCA2a calcium pump. Interleukin-6 was elevated in the presence of myofibroblasts and direct stimulation of hiPSC-CMs with exogenous interleukin-6 recapitulated the paracrine effects of myofibroblasts. Blocking interleukin-6 reduced the effects of myofibroblasts only in the absence of physical contact between cell-types. Myofibroblast-specific connexin43 knockdown reduced functional changes in contact cocultures only when combined with interleukin-6 blockade. This provides the first in-depth investigation into how human myofibroblasts modulate hiPSC-CMs function, identifying interleukin-6 and connexin43 as paracrine- and contact-mediators respectively, and highlighting their potential as targets for reducing arrhythmic risk in cardiac cell therapy.

**Supplementary Information:**

The online version contains supplementary material available at 10.1007/s00018-023-04924-3.

## Introduction

Myocardial infarction (MI), and subsequent heart failure, remain leading causes of mortality and morbidity worldwide. MI leads to cardiomyocyte loss, activation and differentiation of cardiac fibroblasts into myofibroblasts (MyoFBs), and ultimately the formation of a fibrotic scar which often initiates heart failure [1].

Currently available treatments, although capable of slowing disease progression and providing symptomatic relief, are unable to replace the lost myocardium and restore functional contractility. Human pluripotent stem cells (PSCs), such as embryonic stem cells (ESCs) and induced pluripotent stem cells (iPSCs), can generate unlimited numbers of functional cardiomyocytes, providing a promising source for cell-based therapeutic approaches [2]. Transplantation of cardiomyocytes derived from ESCs/iPSCs into the infarcted myocardium of small and large animals can lead to remuscularization and improved cardiac function [3, 4], although long-term engraftment and survival of transplanted cells is questionable [5, 6]. Importantly, in large animals, transplantation of cardiomyocytes is consistently accompanied by ventricular arrhythmias [3, 7], which can be lethal [8]. Recent studies suggest that arrhythmias arise from focally mediated mechanisms originating from the site of transplantation [7, 9], although electrophysiological heterogeneity and conduction slowing or block in regions of engrafted cardiomyocytes have also been proposed to promote vulnerable substrates for re-entry in the post-MI heart [10]. Therefore, arrhythmia has emerged as the major obstacle to the clinical translation of human cardiomyocyte transplantation.

MyoFBs are the predominant cell type in the post-MI infarcted myocardium and drive the healing response and formation of the fibrotic scar [1, 11]. Often characterised by the expression of alpha-smooth muscle actin, these cells exhibit increased secretion of cytokines and soluble factors, specifically TNF-α, IL-6 and IL-1β, in response to pathological stimuli [12, 13]. MyoFBs have also been shown to be persistent, with evidence indicating survival of MyoFBs at the infarct site for up to 17 years in humans [14].

It is important to consider the persistence of MyoFBs in the infarcted myocardium when utilising ESC/iPSC-cardiomyocyte transplantation as a therapeutic option post-MI, as ESC/iPSC-cardiomyocytes are transplanted into a MyoFB-rich environment and remain surrounded by MyoFB-rich fibrotic tissue months after successful engraftment [3, 7]. This is of particular importance as MyoFBs have previously been shown capable of directly modulating the electrophysiology of cardiomyocytes, either through paracrine signalling [15, 16] or direct heterocellular coupling [17–19]. Various paracrine mediators released by MyoFBs have been implicated to affect cardiomyocytes [20, 21], with IL-6, a pro-inflammatory cytokine elevated in MI and congestive heart failure patients, of particular interest through its involvement in cardiac fibrosis and correlation with ventricular dysfunction [22–24]. Gap junctional coupling between cardiomyocytes and cardiac fibroblasts has been demonstrated in vitro [17] and in situ [25], with recent studies showing evidence for cardiomyocyte—non-cardiomyocyte coupling in situ at the ventricular infarct border zone [26, 27] and within the infarct [28] of post-MI mouse hearts. Therefore, MyoFBs present in the infarcted myocardium may affect the therapeutic potential of stem cell therapies post-MI by modulating ESC/iPSC-cardiomyocyte electrophysiology, and potentially play a key role in post-transplantation arrhythmias.

Despite the abundance of MyoFBs residing in the infarcted myocardium, how these cells affect the function, electrophysiology and arrhythmogenic potential of transplanted human iPSC-cardiomyocytes (hiPSC-CMs) remains unknown. In the present study, we tested the hypothesis that adult human cardiac MyoFBs modulate hiPSC-CM action potential and calcium handling, actively contributing to the arrhythmogenic risk of cell therapy. Using a system that allows fine control of cell phenotype and organisation, mimicking one-way/bidirectional paracrine interactions between MyoFBs and transplanted cells prior integration and contact interactions post integration, we; (1) compared the effect of MyoFB paracrine signalling and direct physical contact on hiPSC-CM function; (2) quantified MyoFB-mediated changes to hiPSC-CM ion channel expression; and (3) studied the contribution of specific paracrine signalling and heterocellular coupling to changes in hiPSC-CM function.

## Materials and methods

### Cell culture

Human induced-pluripotent stem cell-derived cardiomyocytes (hiPSC-CMs; iCell Cardiomyocytes—Cellular Dynamics International) were plated onto fibronectin coated (50 µg/mL) glass-bottom dishes, at a density of 6 × 10^4^ cells/dish, as per manufacturer’s protocol. Cells were cultured in cardiomyocytes maintenance media at 37 °C and 5% CO_2_ for 8 days, with medium renewed every 48 h.

Primary human adult ventricular cardiac fibroblasts (ScienCell Research Laboratory) were maintained at 37 °C and 5% CO_2_ and used during experiments between passages 3–5. Fibroblasts were cultured on poly-l-lysine (2 µg/cm^2^) coated culture plates in FM-2 medium containing 5% FBS. Myofibroblasts (MyoFBs) were obtained by activation of fibroblasts through serum-starvation (DMEM medium only) for 12–16 h, followed by treatment with TGF-β1 (5 ng/mL) for 48 h in serum-free DMEM. MyoFBs showed an increased proportion of α-SMA positive cells and increased levels of α-SMA, Col1A1, IL-6 and IL-11 mRNA (Supplementary Fig. 1) compared to control fibroblasts, consistent with a MyoFB phenotype. Flow cytometry further confirmed absence of endothelial and immune cells in MyoFB cultures (Supplementary Fig. 1).

### Coculture setup

**Fig. 1:**
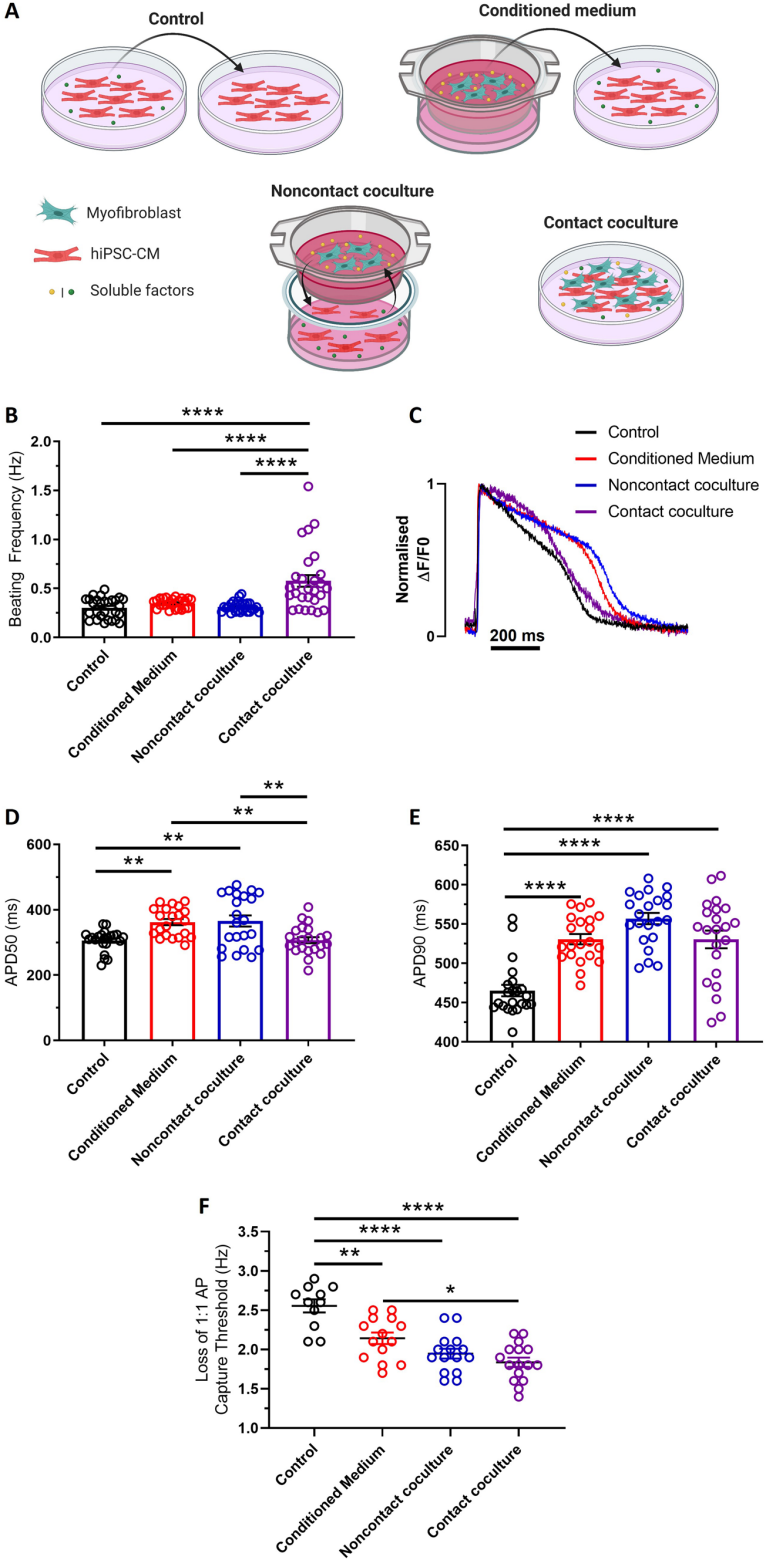
Effects of human cardiac myofibroblasts on hiPSC-CM electrical function. **A** Schematic of culture conditions. hiPSC-CMs were cultured with myofibroblasts (MyoFB) in three conditions: with MyoFB conditioned medium, with MyoFBs separated by a semipermeable membrane (noncontact coculture), and with MyoFBs in direct contact (contact coculture). Control: hiPSC-CM cultured with hiPSC-CM conditioned medium. **B** Spontaneous beating frequency of hiPSC-CM in control, conditioned medium, noncontact and contact coculture (n = 24–28 treated monolayers). **C** Superimposed representative optical action potentials from hiPSC-CM in control (black), conditioned medium (red), noncontact (blue) and contact coculture (purple) conditions. Cells were electrically stimulated at 1 Hz. **D**, **E** Summary of APD50 (**C**) and APD90 (**D**) changes for hiPSC-CM cultured with MyoFBs in the three conditions versus control, at 1 Hz pacing rate (n = 21–22 treated monolayers). **F** Average pacing rate needed to induce loss of 1:1 action potential (AP) capture in hiPSC-CM cultures (n = 11–16 treated monolayers). Bars represent means ± SEM. *p < 0.05, **p < 0.01, ****p < 0.0001 (one‐way ANOVA with post hoc Tukey’s test)

hiPSC-CMs were cultured with adult human cardiac MyoFBs at a ratio of 1:2 for 48 h, in three culture conditions designed to mimic the potential mechanisms of cell–cell interactions (Fig. 1A). To mimic one-way paracrine communication, confluent hiPSC-CM monolayers were treated with media conditioned by MyoFBs (conditioned medium). 1.2 × 10^5^ MyoFBs were cultured in serum-free DMEM for 48 h. Media was collected, centrifuged at 500*g* for 5 min and applied to hiPSC-CMs. To mimic bidirectional paracrine communication whilst preventing direct heterocellular interactions (noncontact coculture), 1.2 × 10^5^ MyoFBs were cultured on 24 mm Transwell^®^ inserts (with 0.4 µm pores) suspended above hiPSC-CMs in serum-free DMEM. To mimic direct heterocellular contact interactions in addition to short-range paracrine communication (contact coculture), 1.2 × 10^5^ MyoFBs were seeded directly on top of hiPSC-CMs in serum-free DMEM. As control, hiPSC-CMs were cultured in the presence of media conditioned by hiPSC-CMs. 6 × 10^4^ hiPSC-CMs were cultured in serum-free DMEM for 48 h. Media was collected and centrifuged at 500*g* for 5 min before application to hiPSC-CM monolayers. Culturing hiPSC-CMs in the absence of serum for 48 h did not affect their electrophysiological properties (Supplementary Fig. 2).

### Voltage and calcium optical mapping

Action potentials (AP) and Ca^2+^ transients were assessed using high resolution optical mapping [29]. Cultures were loaded with FluoVolt (1:1000) or Fluo‐4AM (4 µM plus 1 mM Probenecid). Following dye loading, cultures were transferred to Normal Tyrode’s solution (140 mM NaCl, 4.5 mM KCl, 10 mM Glucose, 10 mM HEPES, 1 mM MgCl_2_, 1.8 mM CaCl_2_, pH 7.4) for optical recordings. For AP recordings, Normal Tyrode’s solution was supplemented with 10 µM blebbistatin to prevent cardiomyocyte movement. Optical mapping was performed with the Photometrics Evolve 512 EMCCD camera (Photometrics) mounted on a custom macroscope (Cairn Research) equipped with a water‐immersion 20× objective, appropriate excitation/emission filters, and 470 nm light‐emitting diode illumination, using the recording software WinFluor [30]. Action potential recordings were performed at 1000 fps, from a 295 × 295 µm area; Ca^2+^ transient recordings were performed at 200 fps, from an 820 × 820 µm area. All recordings were performed at 35–37 °C, with cultures electrically paced at a frequency of 1 Hz (unless otherwise stated). AP and Ca^2+^ transients were analysed using OPTIQ (Cairn Research). For each monolayer culture, multiple regions were recorded per dish (> 5), and for each recording 5 AP/Ca^2+^ transients were analysed, allowing spatial and temporal averaging. Optical mapping signals were filtered using a Gaussian spatial filter (radius 2 pixels) before relevant parameters were extracted [31]. For AP, action potential duration (APD)50 and APD90 were analysed (time from AP upstroke to 50% and 90% repolarisation respectively). For Ca^2+^ transients, time to Ca^2+^ transient peak, time to 50% Ca^2+^ decay (t50) and time to 80% Ca^2+^ decay (t80) were analysed (time from transient peak to 50% and 80% decay respectively). Rate of Ca^2+^ transient decay was calculated by fitting a single exponential equation to the decay phase of each Ca^2+^ transient using Graphpad Prism version 8.1.

### hiPSC-CM spontaneous activity recording

hiPSC-CM spontaneous contractions were recorded using a brightfield microscope immediately prior to dye loading for optical mapping. For each monolayer, three different regions were recorded at 60 fps for ~ 30 s. Spontaneous activity was defined as frequency of contractions (number of contractions/recording time).

### Immunofluorescence staining

Cells were fixed with 4% paraformaldehyde for 10 min at room temperature (RT), permeabilised with 0.1% triton x-100 and blocked with 1% bovine serum albumin (BSA) for 1 h at RT. Cells were incubated with primary antibodies mouse anti-human α-SMA (1:100; Dako) or rabbit anti-Cx43 (1:300; Sigma-Aldrich) overnight at 4 °C, followed by AlexaFluor 488 conjugated secondary antibody (1:200; ThermoFisher) for 2 h at RT. Cells were mounted with VectaShield Propidium iodide. Images were taken with a Nikon Ti-Eclipse A1M confocal microscope using a 20×-objective. Cells positive for α-SMA were counted in 5 randomly chosen images per sample, with a minimum of 100 cells counted per sample. Cx43 was quantified with ImageJ by dividing the area of total Cx43 staining by the number of nuclei in each image.

### Flow cytometry

Fibroblasts and MyoFBs were dissociated using accutase, resuspended in phosphate buffered saline, and viability staining was performed using the Zombie Red Fixable Viability dye (1:100 dilution; BioLegend). Cells were resuspended in cell staining buffer (BioLegend) and Fc receptors were blocked adding TruStain FcX (BioLegend) for 10 min at RT, prior to staining. For cell surface staining, cells were incubated for 30 min at 4 °C in cell staining buffer containing APC/Cy7-conjugated CD31 antibody (BioLegend), Alexa Fluor 700-conjugated CD45 antibody (BioLegend), isotype controls or no antibody. Following incubation, cells were washed with cell staining buffer, and fixed with 4% paraformaldehyde for 15 min at RT. Flow cytometry was performed using the BD FACSCelesta (BD Bioscience) and data analysis was done using FlowJo.

### Fluorescence-activated cell sorting (FACS)

Flow cytometric sorting was used to separate MyoFBs from hiPSC-CMs in contact cocultures, allowing molecular analysis of just hiPSC-CMs. Fibroblasts were stained with CellTrace Far Red (ThermoFisher) prior to MyoFB differentiation as per manufacturer’s instructions. Cells were sorted immediately following optical mapping using the BD FACSAria (BD Bioscience) at 4 °C, with hiPSC-CMs sorted directly into lysis buffer for downstream RNA extraction.

### IL-6 quantification by ELISA

Quantification of IL-6 in media samples collected from different culture conditions was performed using the Human IL-6 Quantikine ELISA kit (R&D systems). Optical density was measured in 96-well plates using a SpectraMax iD3 plate reader (Molecular Devices) at a wavelength of 450 nm, with 540 nm wavelength correction. Standard curves were generated using a 4-parameter logistic curve fit. The standard curve was used to convert the absorbance for each sample into concentration, before final concentrations were calculated by considering any initial dilution factor (1:4 or 1:8 dilution). All assays were done in duplicate. Results are expressed as ng of IL-6/mL media.

### IL-6 stimulation and IL-6 blocking

For IL-6 stimulation experiments, recombinant human IL-6 protein (PHC0064, ThermoFisher) was reconstituted in 100 mM acetic acid to create a 0.1 mg/mL stock solution. Further dilutions were made in a buffer containing 0.1% BSA as carrier protein. hiPSC-CM monolayers were treated with 0 (control), 0.5, 1 and 1.5 ng/mL IL-6 for 48 h.

For experiments where IL-6 signalling was blocked, a human anti-IL-6 neutralising antibody (MA5-23698, ThermoFisher) was used. The antibody concentration was determined by adding increasing concentrations of IL-6 antibody (IL-6 Ab) to hiPSC-CM monolayers stimulated with 1 ng/mL IL-6 (48-h stimulation) to assess which concentration prevented IL-6 induced changes to hiPSC-CM APD and Ca^2+^ decay. The concentrations of IL-6 Ab used were 1 ng/mL, 10 ng/mL and 100 ng/mL, representing 1, 10 and 100 times the molar concentration of IL-6 used. The 100 ng/mL concentration of IL-6 Ab was shown to completely prevent all effects resulting from stimulation of hiPSC-CMs with 1 ng/mL IL-6. During IL-6 neutralising antibody coculture experiments, the IL-6 Ab was added at the point of coculture initiation (following media conditioning) at a concentration of 150 ng/mL, 100 times the highest concentration of IL-6 measured in any coculture sample, to ensure complete IL-6 blockade in all conditions.

### mRNA analysis by Quantitative Real-Time PCR (RT-qPCR)

Total RNA was extracted from hiPSC-CMs or MyoFBs using the RNAqueous™-4PCR Total RNA Isolation Kit (ThermoFisher) as per manufacturer’s instructions. RNA was quantified using the Qubit™ 3.0 Fluorometer (ThermoFisher) and the RNA high sensitivity (HS) assay kit. RNA was reverse transcribed to cDNA using the SuperScript™ VILO™ cDNA Synthesis Kit (ThermoFisher). RT-qPCR was conducted using the ABsolute qPCR ROX Master Mix (ThermoFisher). Samples were analysed on a QuantStudio7 system (Applied Biosystems) in duplicate with Taqman probes. Samples were normalised against the endogenous control gene beta-2-microgloublin (B2M) and analysed using the ΔΔCT method. Taqman assays for RT-qPCR were purchased from Applied Biosystems and are listed in Supplementary Table 1.

### Knockdown of Cx43 by siRNA

siRNA knockdown of Cx43 expression in MyoFBs was performed by transient transfection with two synthetic siRNAs targeting Cx43: siRNA1 (Ambion, s5757), siRNA2 (Ambion, s5759), or a combination of siRNA1 and siRNA2. siRNA targeting GAPDH (Ambion, 4,390,849) was used as a positive control, whilst siRNA that had no significant sequence similarity to human gene sequences (Silencer^®^ Select Negative Control No. 1 siRNA, 4390843) was used as a negative control. BLOCK-iT™ Alexa Fluor™ Red Fluorescent Control (ThermoFisher) was used to check transfection efficiency. MyoFBs in 6-well plates were transfected using Lipofectamine RNAiMAX (ThermoFisher) according to manufacturer’s instructions. Cx43 knockdown was validated using RT-qPCR and Cx43 protein expression assessed with immunostaining analysis.

### Statistical analysis

Student t-test was used to compare values from two groups. One-way ANOVA was used to compare values from more than two groups, with significant group difference compared using Tukey’s multiple comparison post-hoc test. Two-way ANOVA with Tukey’s multiple comparison post-hoc test was used when two variables were present. Statistical analysis was performed using GraphPad Prism version 8.1. Data are presented as mean ± SEM, and a p value of < 0.05 was considered significant.

## Results

### Cardiac myofibroblasts alter hiPSC-CM electrical function and calcium handling

The hypothesis that MyoFBs might affect arrhythmogenicity of hiPSC-CM was investigated by culturing hiPSC-CM with adult human cardiac MyoFBs at a ratio of 1:2, in three culture conditions designed to mimic the potential mechanisms of cell–cell interactions. Confluent hiPSC-CM monolayers were treated with media conditioned by MyoFBs to mimic one-way paracrine communication (conditioned medium); hiPSC-CM monolayers were cocultured with MyoFBs separated by a semipermeable membrane to mimic bidirectional paracrine communication whilst preventing direct heterocellular interactions (noncontact coculture); finally hiPSC-CM monolayers coated with MyoFBs (contact coculture) were used to mimic direct heterocellular contact interactions in addition to short-range paracrine communication. hiPSC-CMs were cultured in the presence of hiPSC-CM-conditioned medium as control condition (Fig. 1A).

**Fig. 2:**
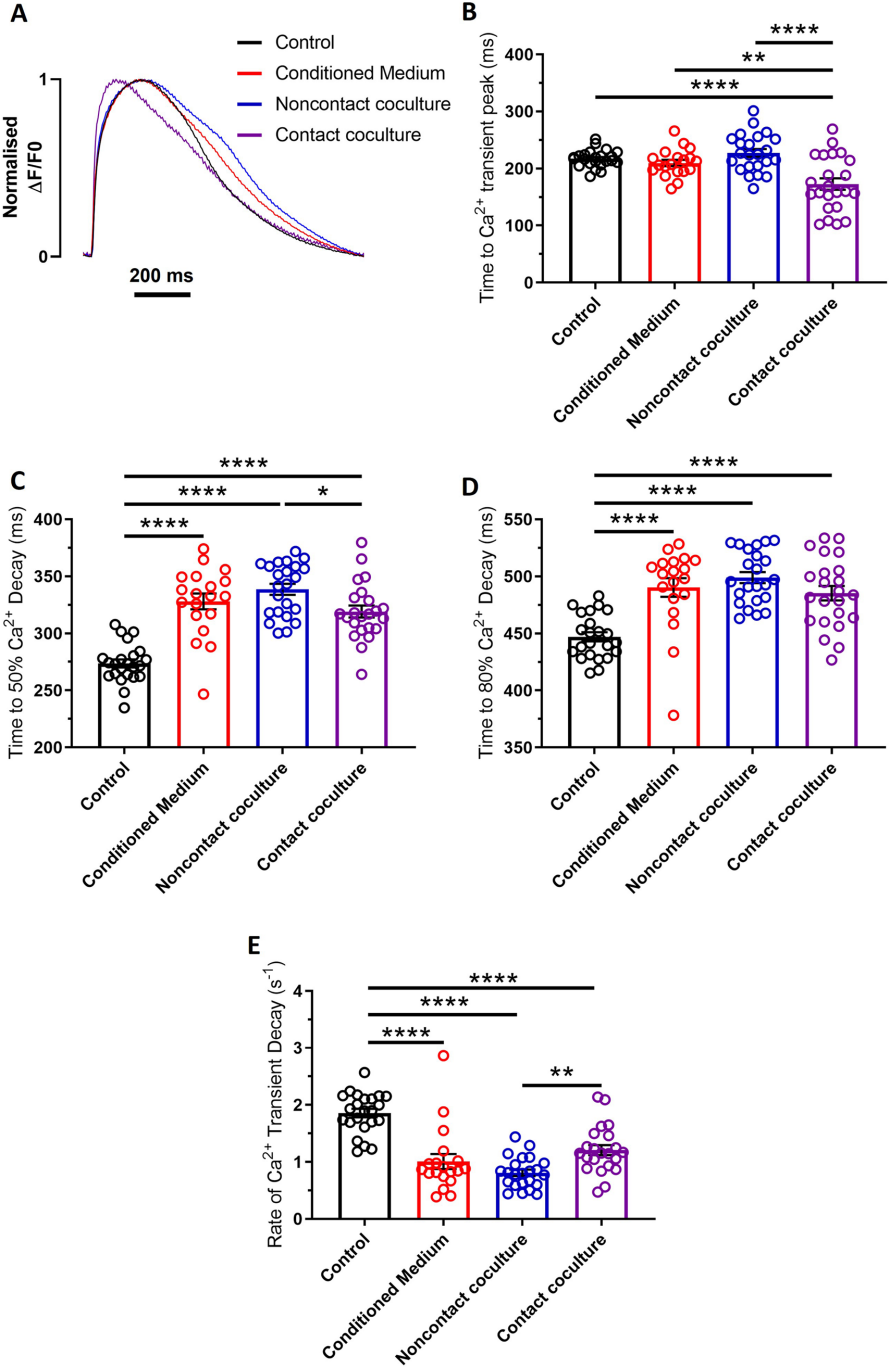
Effects of human cardiac myofibroblasts on hiPSC-CM calcium handling. **A** Superimposed representative optical Ca^2+^ transients from hiPSC-CM in control (black), conditioned medium (red), noncontact (blue) and contact coculture (purple) conditions. Cells were electrically stimulated at 1 Hz. **B**–**E** Summary of time to Ca^2+^ transient peak (**B**), time to 50% Ca^2+^ decay (**C**), time to 80% Ca^2+^ decay (**D**), and rate of Ca^2+^ transient decay (**E**) changes for hiPSC-CM cultured with MyoFBs in the three conditions versus control, at 1 Hz pacing rate. Bars represent means ± SEM (n = 12–23 treated monolayers). *p < 0.05, **p < 0.01, ****p < 0.0001 (one‐way ANOVA with post hoc Tukey’s test)

After 48 h of culture, hiPSC-CMs were assessed for several electrophysiological and calcium handling parameters using brightfield microscopy and optical mapping (Figs. 1 and 2). Spontaneous beating frequency was significantly increased in contact coculture versus control but was unaffected by conditioned medium and in noncontact coculture (Fig. 1B). Optical action potentials (AP) recorded at 1 Hz pacing rate (Fig. 1C) revealed that hiPSC-CM APD50 increased with conditioned medium treatment and in noncontact cocultures compared to control but did not significantly change in contact cocultures (Fig. 1D). However, APD90 was prolonged in all culture conditions compared to control, with the largest effect observed in noncontact cocultures (+ 19.7% versus control; Fig. 1E). AP recorded at progressively faster pacing rates revealed that loss of 1:1 AP capture occurred at lower threshold in conditioned medium (2.14 ± 0.08 Hz, p < 0.01), noncontact (1.95 ± 0.07 Hz, p < 0.0001) and contact coculture (1.84 ± 0.06 Hz, p < 0.0001) compared to control (2.56 ± 0.08 Hz; Fig. 1F).

Optical Ca^2+^ transients recorded at 1 Hz pacing rate (Fig. 2A) were also affected by MyoFB coculture. Time to Ca^2+^ transient peak was reduced following contact coculture (− 20.33% versus control, p < 0.0001; Fig. 2B), but unaffected in other culture conditions. Time to 50% Ca^2+^ decay (t50) (Fig. 2C) and time to 80% Ca^2+^ decay (t80) (Fig. 2D) were prolonged in all culture conditions compared to control. Also, the rate of Ca^2+^ transient decay was reduced in all culture conditions compared to control (Fig. 2E).

### Cardiac myofibroblasts alter the abundance of mRNAs encoding ion channels in hiPSC-CM

**Fig. 3:**
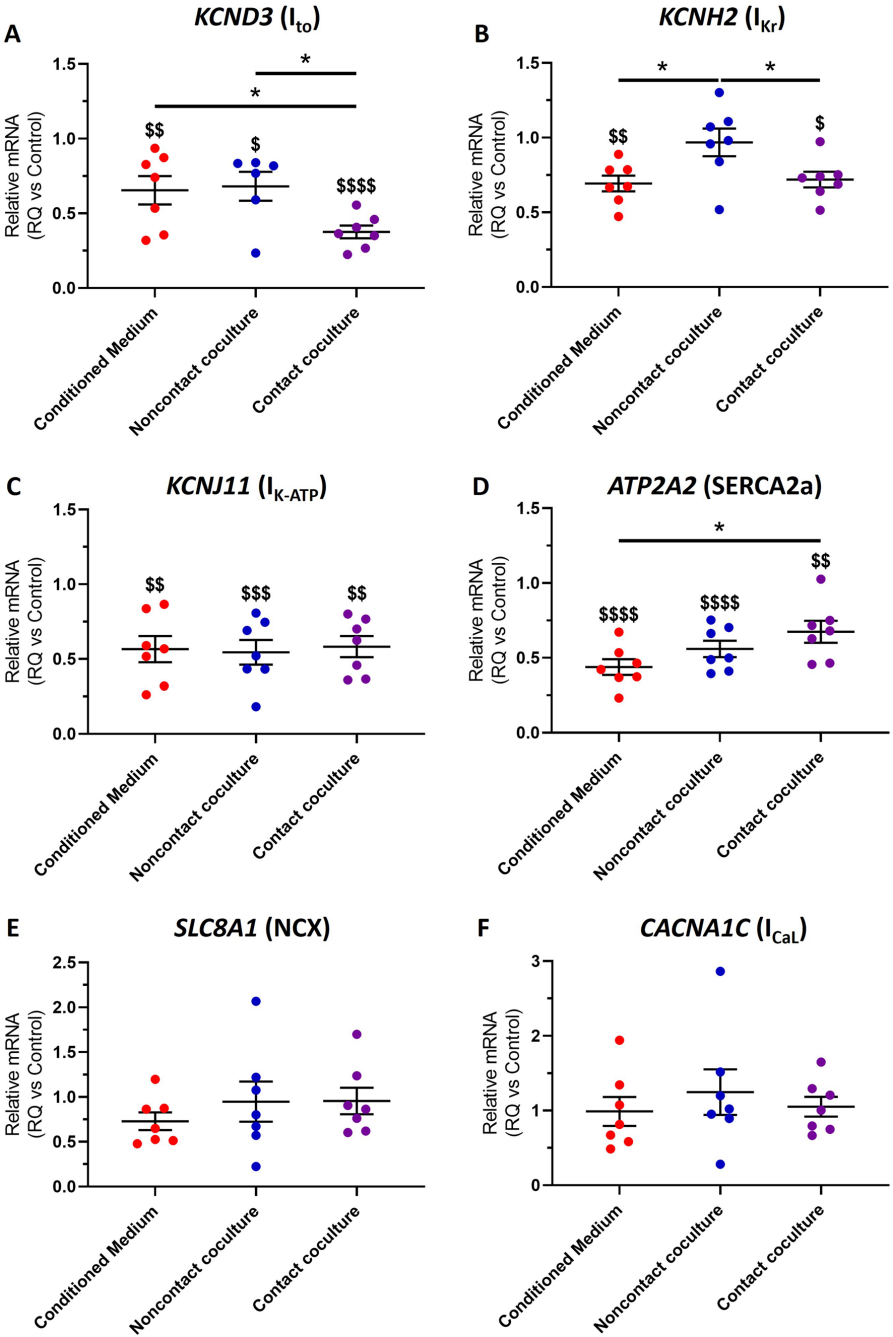
Effect of human cardiac myofibroblasts on hiPSC-CM molecular profile. **A**–**F** Changes in the abundance of mRNAs associated with AP repolarisation and Ca^2+^ cycling in hiPSC-CM after culture with MyoFBs conditioned medium (red), with MyoFBs separated by a semipermeable membrane (noncontact coculture, blue), and with MyoFBs in direct contact (contact coculture, purple). Levels of each mRNA were normalised to the control group (hiPSC-CM cultured in the presence of hiPSC-CM-conditioned medium; RQ = 1 for all genes). *KCND3* encodes the transient outward potassium channel K_V_4.3; *KCNH2* encodes the potassium voltage-gated channel K_V_11.1; *KCNJ11* encodes the Kir6.2 protein which contributes to I_K-ATP_; *ATP2A2* encodes the sarcoplasmic reticulum Ca^2+^-ATPase (SERCA2a); *SLC8A1* encodes the sodium-calcium exchanger (NCX1); *CACNA1C* encodes Ca_V_1.2 protein which contributes to I_CaL_. RT-qPCR performed on mRNA isolated from hiPSC-CM only. Values expressed as means ± SEM (n = 6–7 treated monolayers). ^$^p < 0.05, ^$$^p < 0.01, ^$$$^p < 0.001, ^$$$$^p < 0.0001 versus control. *p < 0.05 (one‐way ANOVA with post hoc Tukey’s test)

To investigate the molecular mechanism underlying the changes in hiPSC-CM electrical function and Ca^2+^ handling following MyoFB coculture, we quantified changes in the abundance of mRNAs associated with AP repolarisation and Ca^2+^ cycling in hiPSC-CMs. For contact cocultures, hiPSC-CMs were separated from MyoFBs using FACS before mRNA extraction. Levels of each mRNA tested were normalised to the control group (hiPSC-CM cultured in the presence of hiPSC-CM-conditioned medium; RQ = 1 for all genes; Fig. 3). As shown in Fig. 3A, the mRNA levels of *KCND3* (encodes the K_V_4.3 protein) were reduced in all culture conditions compared to control, and were significantly lower in contact coculture compared to both conditioned medium and noncontact coculture. The mRNA levels of *KCNH2* (encodes the K_V_11.1 protein) were significantly reduced in conditioned medium and contact coculture, but were unchanged in noncontact coculture (Fig. 3B). Furthermore, the mRNA levels of *KCNJ11* (encodes the Kir6.2 protein) were also significantly reduced in all culture conditions (Fig. 3C). *ATP2A2* (encodes SERCA2a) mRNA levels were reduced in all culture conditions (Fig. 3D), however *SLC8A1* (encodes the sodium-calcium exchanger—NCX1; Fig. 3E) and *CACNA1C* (encodes Ca_V_1.2 protein which contributes to I_CaL_; Fig. 3F) mRNA levels remained unchanged in all conditions.

### Stimulation of hiPSC-CMs with IL-6 recapitulates the paracrine-mediated effects of cardiac myofibroblasts

IL-6 was measured in the media collected from the different culture conditions by ELISA. IL-6 was detected at very low levels in hiPSC-CM control cultures (< 0.001 ng/mL) but was significantly increased in all MyoFB-treated cultures (conditioned medium: 0.4 ± 0.06 ng/mL; noncontact: 0.5 ± 0.1 ng/mL; contact: 1 ± 0.1 ng/mL; p < 0.05, p < 0.01, p < 0.0001 respectively; Supplementary Fig. 3). Importantly, RT-qPCR analysis revealed that hiPSC-CMs express mRNA encoding the interleukin-6 receptor (IL-6R), which we show remains unchanged during stimulation with different concentrations of exogenous IL-6 (Supplementary Fig. 4F).

Stimulation of hiPSC-CMs with exogenous IL-6 was able to recapitulate the paracrine-mediated effects of MyoFB on hiPSC-CM function. Treatment of hiPSC-CMs with 0.5, 1 and 1.5 ng/mL (to cover the range of IL-6 concentrations observed in different culture conditions) for 48 h led to APD50 and APD90 prolongation, whilst 1 and 1.5 ng/mL also led to loss of 1:1 AP capture at significantly lower pacing rates (Supplementary Fig. 3). Exogenous IL-6 treatment prolonged t50 and t80, and reduced rate of Ca^2+^ transient decay (Supplementary Fig. 4). As expected, no concentration-depended effect was observed as a narrow range of IL-6 concentrations was used (0.5–1.5 ng/mL). Importantly, exogenous IL-6 effects were similar in magnitude to those observed in conditioned medium and noncontact cocultures (Figs. 1 and 2).

### Blockade of IL-6 signalling reduces the effect of paracrine-mediated communication during myofibroblast-hiPSC-CM coculture, but has no effect on direct contact coculture

To further investigate the role of IL-6, we blocked IL-6 signalling in all culture conditions by adding an anti-IL-6 neutralising antibody (IL-6 Ab) to the culture medium for the 48 h coculture period. The concentration of IL-6 Ab used was 150 ng/mL (~ 100 times the highest concentration of IL-6 measured in the media of all culture conditions) as this concentration completely prevented the APD and t50 prolongation observed during stimulation of hiPSC-CM by exogenous IL-6 (Supplementary Fig. 5).

**Fig. 4:**
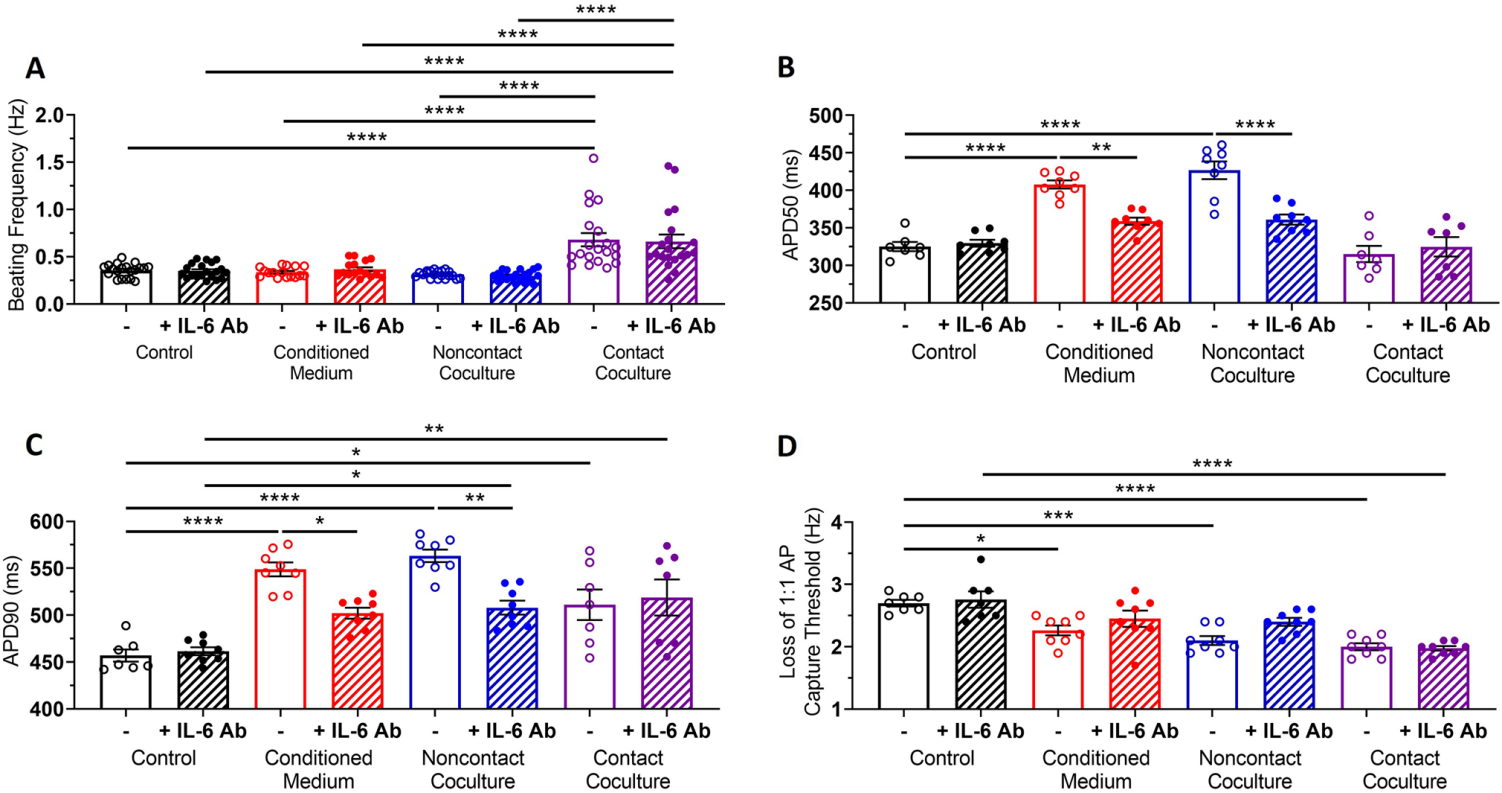
Blockade of IL-6 signalling reduces the effects of myofibroblast-conditioned medium and noncontact coculture on hiPSC-CM electrical function. **A** Spontaneous beating frequency of hiPSC-CM in control (black), conditioned medium (red), noncontact (blue) and contact coculture (purple), in the presence (diagonal pattern) or absence (no pattern) of a neutralising IL-6 antibody. Values expressed as means ± SEM (n = 16–20 treated monolayers). **B**, **C** Summary of APD50 (**B**) and APD90 (**C**) for hiPSC-CM in control, conditioned medium, noncontact and contact coculture, in the presence (diagonal pattern) or absence (no pattern) of a neutralising IL-6 antibody, at 1 Hz pacing rate. Values expressed as means ± SEM (n = 7–8 treated monolayers). **D** Average pacing rate needed to induce loss of 1:1 action potential capture in hiPSC-CM cultures. Values expressed as means ± SEM (n = 7–8 treated monolayers). *p < 0.05, **p < 0.01, ***p < 0.001, ****p < 0.0001 (two‐way ANOVA with post hoc Tukey’s test)

Addition of the IL-6 Ab did not block the increase in hiPSC-CM spontaneous beating frequency observed in contact coculture (Fig. 4A). However, addition of the IL-6 Ab prevented APD50 prolongation (Fig. 4B) and reduced APD90 prolongation (Fig. 4C) in hiPSC-CM cultures treated with conditioned medium and in noncontact cocultures but had no effect on APD90 prolongation observed in contact cocultures (Fig. 4C). Addition of the IL-6 Ab prevented changes in loss of 1:1 AP capture threshold following treatment of hiPSC-CMs with conditioned medium or noncontact coculture but did not prevent threshold changes in contact cocultures (Fig. 4D).

**Fig. 5:**
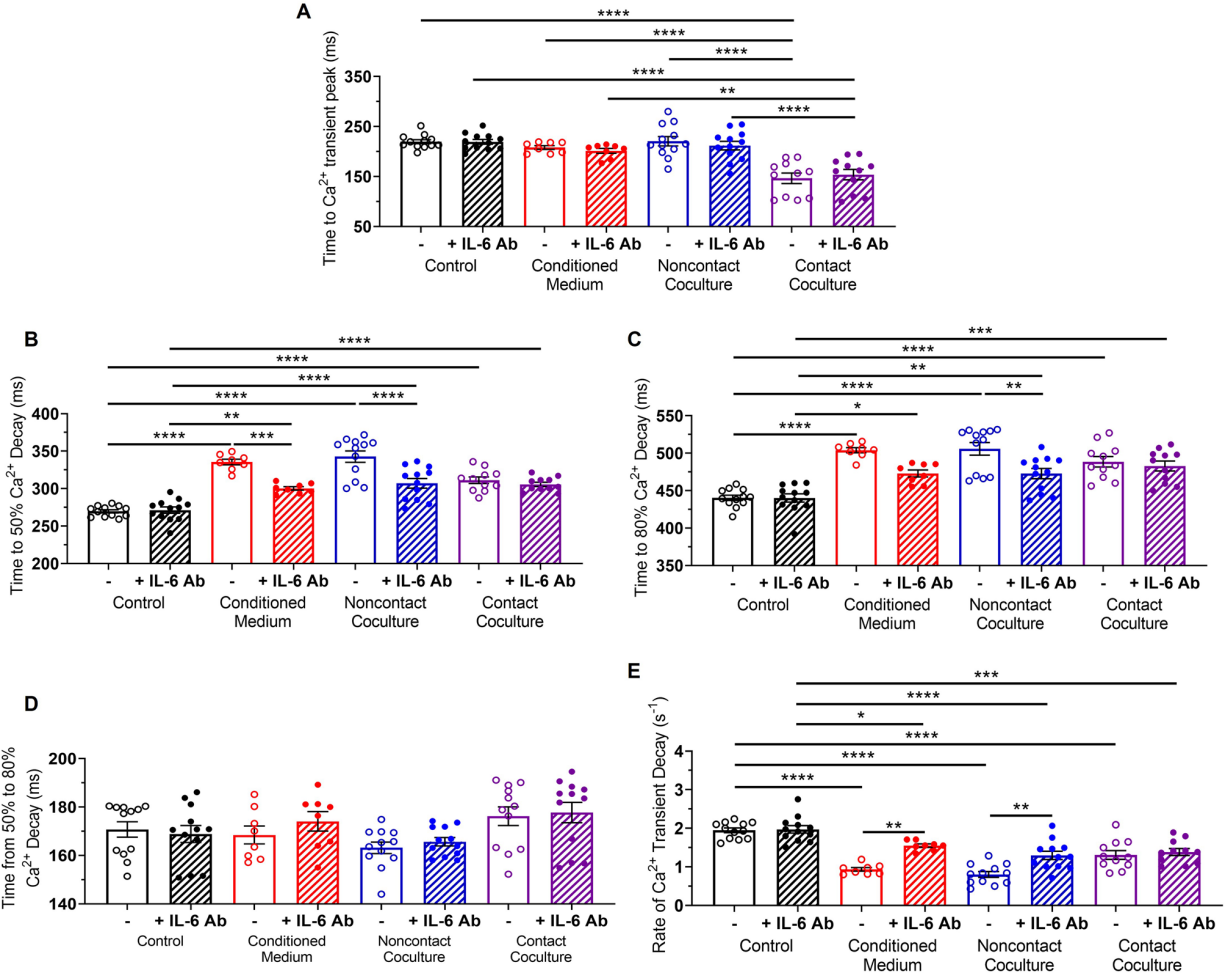
Blockade of IL-6 signalling reduces the effects of myofibroblast-conditioned medium and noncontact coculture on hiPSC-CM calcium handling. **A–E** Time to Ca^2+^ transient peak (**A**), time to 50% Ca^2+^ decay (**B**), time to 80% Ca^2+^ decay (**C**), time from 50 to 80% Ca^2+^ decay (**D**) and rate of Ca^2+^ transient decay (**E**) for hiPSC-CM in control (black), conditioned medium (red), noncontact (blue) and contact coculture (purple), in the presence (diagonal pattern) or absence (no pattern) of a neutralising IL-6 antibody, at 1 Hz pacing rate. Values expressed as means ± SEM (n = 8–12 treated monolayers). *p < 0.05, **p < 0.01, ***p < 0.001, ****p < 0.0001 (two‐way ANOVA with post hoc Tukey's test)

Addition of the IL-6 Ab reduced the increase in hiPSC-CM t50 and t80 in conditioned medium and noncontact cocultures, but did not affect the prolonged Ca^2+^ transient decay in contact cocultures (Fig. 5B, C) or prevent the reduction in time to Ca^2+^ transient peak observed in contact cocultures (Fig. 5A). Late-phase Ca^2+^ decay, the time from t50 to t80, was unaffected in all culture conditions and did not change with addition of IL-6 Ab (Fig. 5D). Further, addition of the IL-6 Ab reduced the effect of conditioned medium and noncontact coculture on hiPSC-CM rate of Ca^2+^ decay (Fig. 5E).

These results indicate IL-6 plays a key role in the electrophysiological and Ca^2+^ handling changes observed in cultures mimicking paracrine interactions between MyoFBs and hiPSC-CMs in the absence of contact interactions.

### Connexin43 knockdown in myofibroblasts does not prevent electrophysiological and calcium handling changes in direct contact coculture

Connexin43 (Cx43) has previously been implicated in gap-junction mediated heterocellular coupling between fibroblasts and cardiomyocytes both in vitro and in situ [32]. MyoFBs at the infarct border zone and in the scar express Cx43 [17, 27, 28, 33] and can electrically couple with cardiomyocytes [26–28]. This functional coupling mediated by Cx43 interactions has been proposed to promote arrhythmogenic phenotypes [28, 34].

**Fig. 6:**
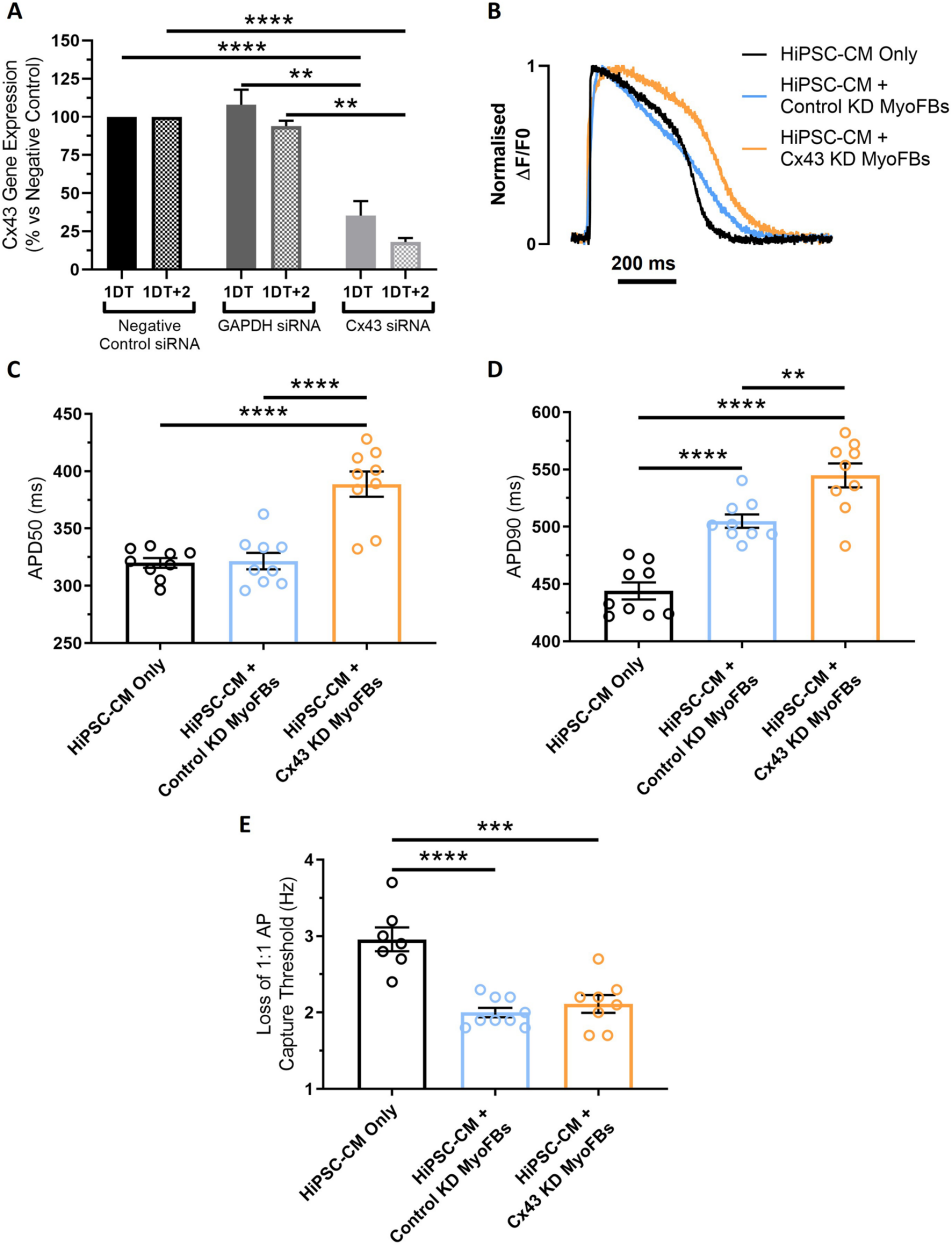
Effect of Cx43 silencing in myofibroblasts on hiPSC-CM electrical function following contact coculture. **A** Quantification of changes in Cx43 mRNA abundance in MyoFBs following transfection with a negative control siRNA, a GAPDH-specific siRNA, and a Cx43-specific siRNA (Ambion Silencer™ Select siRNA s5759) by RT-qPCR. RNA was collected at two timepoints: immediately following transfection (24-h transfection time; 1DT), and 48 h after transfection was completed (cells washed post-transfection and then cultured in basal DMEM; 1DT + 2). Values expressed as percentage relative to the negative control ± SEM (n = 3 independent experiments). **p < 0.01, ****p < 0.0001 (one‐way ANOVA with post hoc Tukey's test). **B** Superimposed representative optical AP from hiPSC-CM cultured in direct contact with MyoFBs treated with a non-targeting negative control siRNA (HiPSC-CM + Control KD MyoFBs, light blue) or in contact with MyoFBs treated with a Cx43-specific siRNA (HiPSC-CM + Cx43-KD MyoFBs, orange). Pure hiPSC-CM cultures (HiPSC-CM Only, black): hiPSC-CM cultured with basal DMEM media. Cells were electrically stimulated at 1 Hz. **C**, **D** APD50 (**C**) and APD90 (**D**) changes for hiPSC-CM cultured in contact with control KD and Cx43-KD MyoFBs versus hiPSC-CM alone, at 1 Hz pacing rate. Values expressed as means ± SEM (n = 9 treated monolayers). **E** Average pacing rate needed to induce loss of 1:1 action potential capture in hiPSC-CM cultures. Values expressed as means ± SEM (n = 7–9 treated monolayers). **p < 0.01, ***p < 0.001, ****p < 0.0001 (one‐way ANOVA with post hoc Tukey's test)

As IL-6 blockade did not prevent the electrical and Ca^2+^ handling changes observed in contact coculture, we decided to assess the contribution of Cx43-mediated interactions by downregulating Cx43 expression specifically in MyoFBs, prior to culture in contact with hiPSC-CMs. Knockdown of Cx43 was achieved by transfecting MyoFBs with a Cx43-specific siRNA (Ambion Silencer™ Select siRNA s5759) and was compared against MyoFBs transfected with a negative control siRNA (Ambion Silencer™ Select Negative Control No. 1 siRNA) and GAPDH-specific siRNA (Ambion Silencer™ Select GAPDH Positive Control siRNA). Knockdown of Cx43 mRNA was confirmed via RT-qPCR, with significant knockdown observed both immediately following transfection (1 day of transfection, 1DT; 64.7% knockdown; p < 0.01 versus negative control) and 48 h-post transfection (1DT + 2; 81.9% knockdown; p < 0.0001 versus negative control), confirming Cx43 mRNA knockdown (Cx43-KD) remained for the duration of the contact coculture (Fig. 6A). No change in Cx43 mRNA levels were detected following transfection with GAPDH-specific siRNA at 1DT or 1DT + 2 (Fig. 6A). RT-qPCR data were complemented with immunostaining analysis, showing Cx43 protein expression was significantly lower in Cx43-KD MyoFBs compared to control cells (Supplementary Fig. 6). MyoFBs transfected with the negative control siRNA (control KD MyoFBs) and non-transfected MyoFBs had the same effect on hiPSC-CM APD and Ca^2+^ transients (Supplementary Fig. 7).

Following knockdown of Cx43 in MyoFBs, optical AP were recorded at 1 Hz pacing rate in hiPSC-CMs alone, in contact with control KD MyoFBs, or in contact with Cx43-KD MyoFBs (Fig. 6B). Cx43-KD did not prevent hiPSC-CM APD90 prolongation observed in cocultures with control KD MyoFBs compared to hiPSC-CMs alone, but further exacerbated the effect (Fig. 6D). Furthermore, Cx43-KD MyoFBs significantly prolonged APD50 compared to both control KD MyoFBs and hiPSC-CMs alone (Fig. 6C). Cx43-KD did not prevent the increased susceptibility to loss of 1:1 AP capture observed in cocultures with control KD MyoFBs (Fig. 6E).

**Fig. 7:**
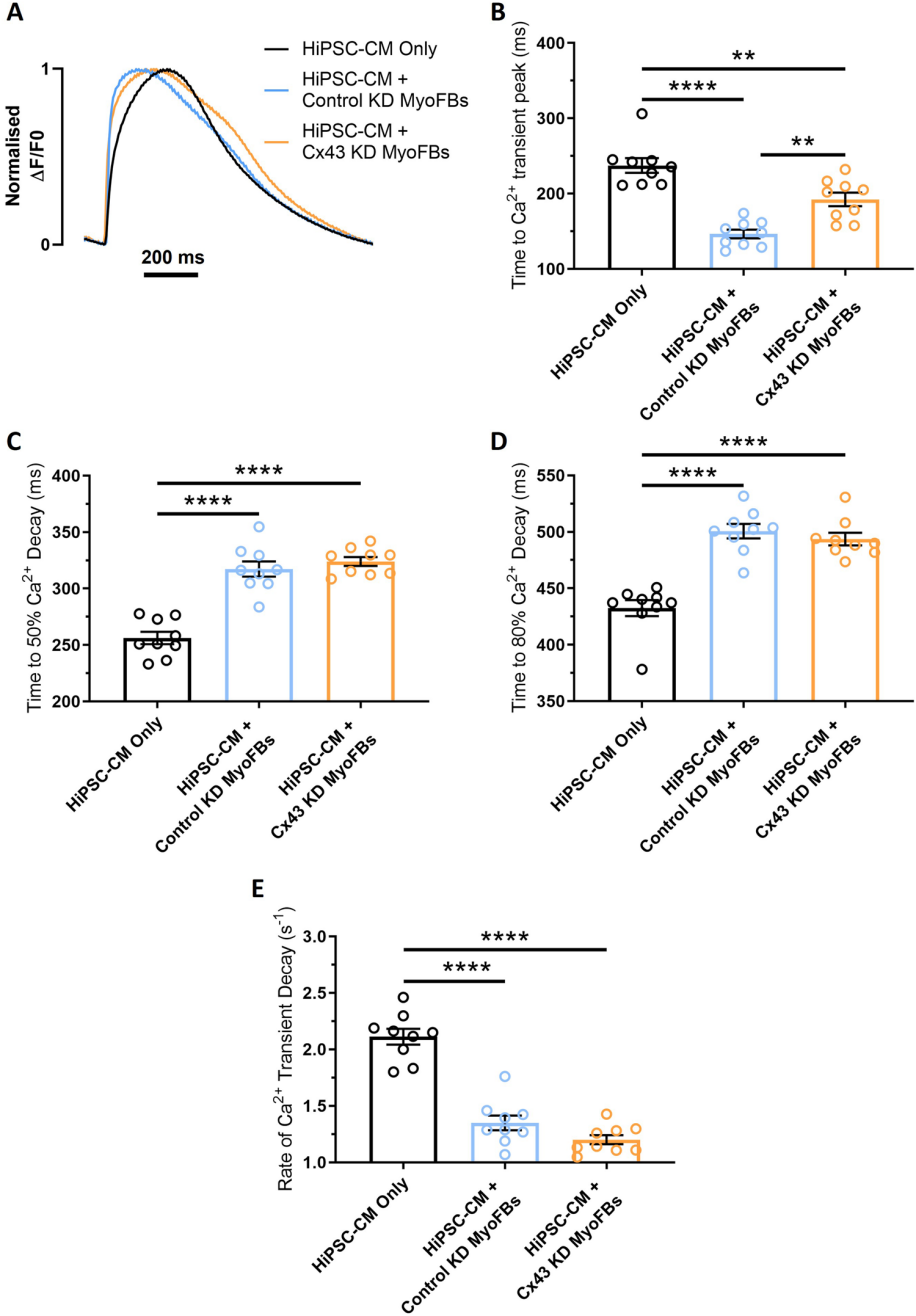
Effect of Cx43 silencing in myofibroblasts on hiPSC-CM calcium handling following contact coculture. **A** Superimposed representative optical Ca^2+^ transients from hiPSC-CM cultured in direct contact with MyoFBs treated with a non-targeting negative control siRNA (HiPSC-CM + Control KD MyoFBs, light blue) or in contact with MyoFBs treated with a Cx43-specific siRNA (HiPSC-CM + Cx43-KD MyoFBs, orange). Pure hiPSC-CM cultures (HiPSC-CM Only, black): hiPSC-CM cultured with basal DMEM media. Cells were electrically stimulated at 1 Hz. **B**–**E** Summary of time to Ca^2+^ transient peak (**B**), time to 50% Ca^2+^ decay (**C**), time to 80% Ca^2+^ decay (**D**) and rate of Ca^2+^ transient decay (**E**) changes for hiPSC-CM cultured in contact with control KD MyoFBs or Cx43-KD MyoFBs versus hiPSC-CM alone, at 1 Hz pacing rate. Bars represent means ± SEM (n = 9 treated monolayers). **p < 0.01, ****p < 0.0001 (one‐way ANOVA with post hoc Tukey's test)

Similarly, optical Ca^2+^ transients were recorded at 1 Hz pacing rate in hiPSC-CMs alone, in contact with control KD MyoFBs, or in contact with Cx43-KD MyoFBs (Fig. 7A). Cx43-KD in MyoFBs did not prevent or reduce the increase in hiPSC-CM t50 or t80 observed in cultures with control KD MyoFBs (Fig. 7C, D), however Cx43-KD significantly reduced the decrease in time to Ca^2+^ transient peak observed in cultures with control KD MyoFBs versus hiPSC-CMs alone (Fig. 7B). Cx43-KD MyoFBs did not prevent rate of Ca^2+^ transient decay reduction (Fig. 7E).

These results indicate that knockdown of Cx43 in MyoFBs is unable to prevent hiPSC-CM electrophysiological and Ca^2+^ handling changes observed when the two cell types are cultured in contact. On the contrary, knockdown of Cx43 in MyoFBs promotes further changes in APD50 and APD90, similar to those observed in noncontact cocultures.

### Combination of myofibroblast-Cx43 knockdown and IL-6 blockade reduces electrophysiological and calcium handling changes in direct contact coculture

As knockdown of Cx43 in MyoFBs in contact cocultures appeared to promote changes in hiPSC-CM function similar to those observed in noncontact cocultures, we combined MyoFB-specific Cx43-KD with IL-6 neutralisation to quantify the contribution of IL-6 signalling in this setting.

**Fig. 8:**
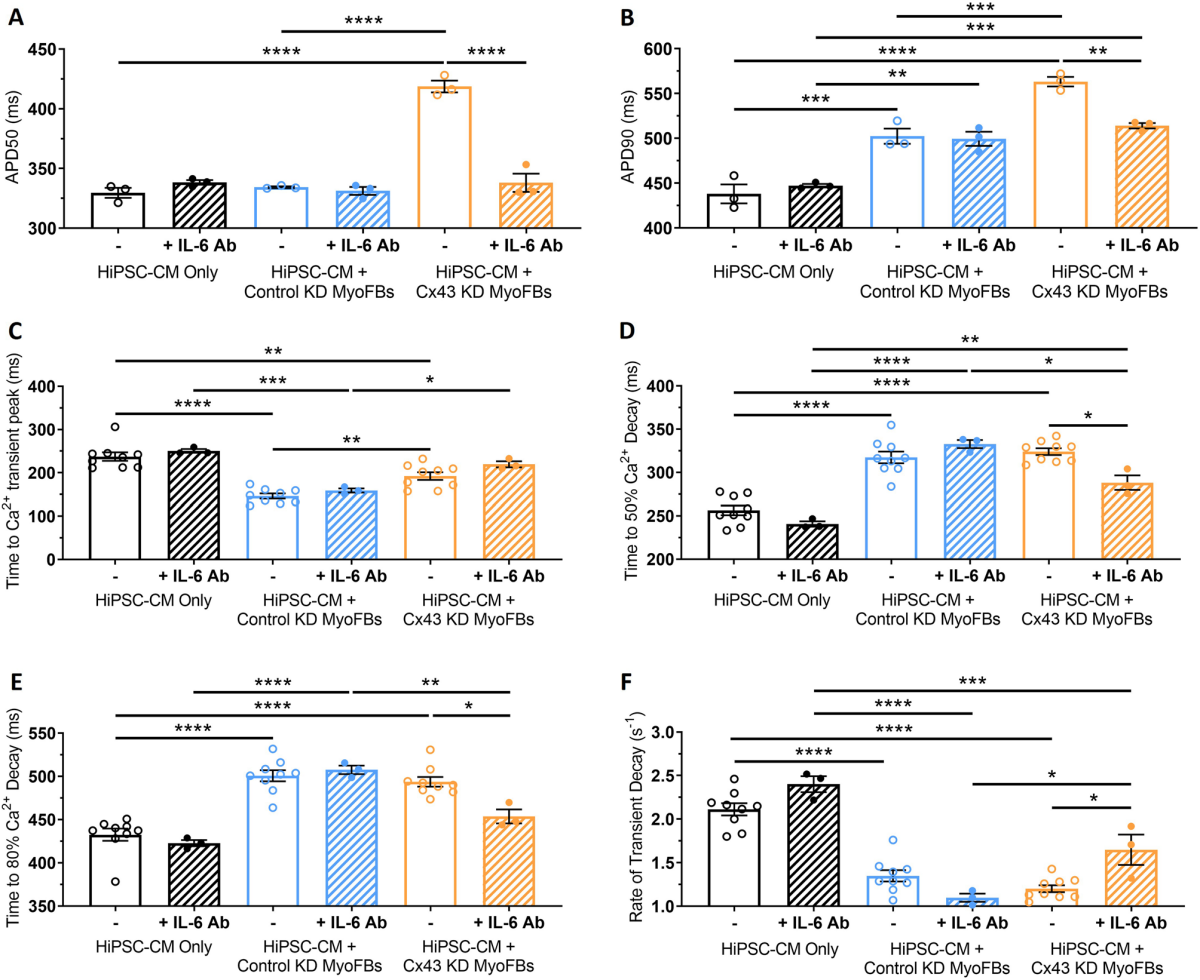
Effect of myofibroblast Cx43 silencing and IL-6 signalling blockade on hiPSC-CM electrical function and calcium handling following contact coculture. **A**–**F** APD50 (**A**), APD90 (**B**), time to Ca^2+^ transient peak (**C**), time to 50% Ca^2+^ decay (**D**), time to 80% Ca^2+^ decay (**E**) and rate of Ca^2+^ transient decay (**F**) for hiPSC-CM cultured in contact with control KD MyoFBs or Cx43-KD MyoFBs versus hiPSC-CM alone, in the presence (diagonal pattern) or absence (no pattern) of a neutralising IL-6 antibody, at 1 Hz pacing rate. Values expressed as means ± SEM (n = 3–9 treated monolayers). *p < 0.05, **p < 0.01, ***p < 0.001, ****p < 0.0001 (two‐way ANOVA with post hoc Tukey’s test)

Optical APs recorded at 1 Hz pacing rate showed that treating Cx43-KD MyoFB—hiPSC-CM contact cocultures with IL-6 Ab prevented APD50 (Fig. 8A) and reduced APD90 prolongation (Fig. 8B), recapitulating effects observed when treating noncontact cocultures with IL-6 Ab (Fig. 4B, C).

Optical Ca^2+^ transients recorded at 1 Hz pacing rate showed that combination of IL-6 blockade and MyoFB-specific Cx43-KD prevented the reduction in hiPSC-CM time to Ca^2+^ transient peak (Fig. 8C) and the increase in t80 (Fig. 8E), and reduced the increase in t50 (Fig. 8D) observed in control KD MyoFBs contact cocultures versus hiPSC-CMs alone. Further, combination of IL-6 Ab with Cx43-KD reduced the effect of contact coculture on hiPSC-CM rate of Ca^2+^ decay (Fig. 8F). In control contact cocultures, addition of the IL-6 Ab had no effect on hiPSC-CM time to Ca^2+^ transient peak, t50, t80 or rate of Ca^2+^ transient decay (Fig. 8C–F), consistent with results in Figs. 4 and 5.

These results indicate IL-6 plays a major role in contact cocultures following MyoFB-specific Cx43 knockdown, suggesting Cx43 may suppress or prevent IL-6 paracrine signalling during contact cocultures.

## Discussion

Improved understanding of how grafted cell therapies interact with the native myocardium is essential for treatment optimisation and prevention of potentially fatal arrythmia. Here we shed light on the mechanisms through which MyoFBs, the predominant cell-type in the recipient tissue, modulate the electrophysiology and Ca^2+^ handling of hiPSC-CMs and identify potential targets for reducing arrhythmic risk in cardiac cell therapy. The key findings are that (1) human adult cardiac MyoFBs exert adverse effects on the electrophysiological and Ca^2+^ handling properties of hiPSC-CMs through specific paracrine-mediated and direct heterocellular coupling mechanisms; (2) blocking IL-6 signalling prevents/reduces changes observed in paracrine-mediated cultures; (3) MyoFB-specific Cx43 knockdown alone does not prevent changes in contact cocultures, but combined Cx43 knockdown and IL-6 blockade has beneficial effects. To our knowledge, this is the first study to perform in-depth investigation of changes to hiPSC-CM AP and Ca^2+^ transients following coculture with adult human MyoFBs through both paracrine- and contact-mediated signalling.

The results presented here show that MyoFBs prolong hiPSC-CM APD50, APD90 and Ca^2+^ transient decay, with no changes in time to Ca^2+^ transient peak, when cocultured in a system that only allows paracrine communication. Furthermore, treating hiPSC-CM with MyoFBs-conditioned medium displays similar effects, suggesting that a dynamic interaction between the two cell types is not required to promote these functional changes. MyoFBs paracrine factors have been shown to prolong APD in neonatal and adult rat cardiomyocytes [15, 16, 35] and mouse ESC-cardiomyocytes [36], although specific paracrine factors and signalling mechanisms are yet to be identified. The molecular mechanisms underlying APD prolongation in these settings also remain poorly defined. Previous work indicates that soluble mediators released by MyoFBs may alter transcription of ion channels in neonatal and adult rat cardiomyocytes [15, 16, 35]. In this study we report reduced *KCND3*, *KCNH2*, and *KCNJ11* mRNA expression in hiPSC-CMs, with these genes encoding the pore-forming subunits of I_to_, I_Kr_ and I_K-ATP_ channels, respectively. Downregulation of *KCND3/KCND2* and *KCNJ11* mRNA, as well as inhibition of I_to_, I_Kr_ and I_K-ATP_ currents have been associated with prolonged APD [15, 16, 35, 37–39]. While the reduction in *KCND3*, *KCNH2*, and *KCNJ11* mRNA levels could account for the observed APD50 and APD90 prolongation in our study, changes in the expression of channel-interacting proteins (e.g. KChIP2 [16]), other ion channels (e.g. I_Ks_) or post-translational modifications (e.g. phosphorylation) cannot be excluded as contributors. Importantly, no changes in *SLC8A1* and *CACNA1C* mRNA levels were detected in our hiPSC-CM cultures, indicating that L-type calcium channels and sodium-calcium exchanger (NCX) are unlikely to contribute to APD prolongation.

The effect of MyoFBs paracrine factors on cardiomyocyte Ca^2+^ handling remains largely unexplored. In an earlier study, Cartledge et al. [20] showed that soluble factors released by adult rat MyoFBs reduce Ca^2+^ transient decay in adult rat cardiomyocyte. We report prolongation of Ca^2+^ transient decay in hiPSC-CMs treated with adult human MyoFBs-conditioned medium and in noncontact cocultures. The molecular mechanisms underlying MyoFBs-mediated Ca^2+^ handling changes in cardiomyocytes have not been previously investigated. The prolonged Ca^2+^ decay observed in our study is likely a result of the reduced *SERCA2a* mRNA expression, with SERCA2a the primary route of Ca^2+^ removal in hiPSC-CMs [40]. This is further supported by our observation that early Ca^2+^ decay (t50) was significantly prolonged, but late Ca^2+^ decay (t50-t80) was unaffected by MyoFBs, with early Ca^2+^ removal mainly driven via SERCA2a, and late phase Ca^2+^ removal primarily driven via NCX [41].

The effect of heterocellular contact interactions on cardiomyocyte AP remains controversial, with studies performed on animal cells suggesting both APD shortening [17, 42] and prolongation [17, 34, 43]. Our results demonstrate that adult human MyoFBs prolong hiPSC-CM APD90, but have no effect on APD50, when cocultured in direct physical contact. qRT-PCR analysis of hiPSC-CMs shows downregulation of *KCND3*, *KCNH2*, and *KCNJ11* mRNA. Given the role of I_to_ current in early AP repolarisation, the reduced *KCND3* mRNA expression following contact coculture would be expected to prolong APD50, as we observed in noncontact cocultures. The gap junction current (I_Gap_) identified in computational studies of fibroblasts coupled with cardiomyocytes could explain this difference [44]. The early I_to_-like component of I_Gap_ could generate transient outward current counteracting the effect of reduction in *KCND3* mRNA, while the late background current component could act as current source during late repolarisation to prolong APD90 [44–46]. Downregulation of *KCNH2* and *KCNJ11* mRNA is also likely to contribute to APD90 prolongation.

Direct contact between human MyoFBs and hiPSC-CMs also altered Ca^2+^ handling, significantly prolonging Ca^2+^ transient decay and reducing time to Ca^2+^ transient peak. The reduced *SERCA2a* mRNA expression could account for the early Ca^2+^ decay prolongation, however the mechanism behind the reduced time to Ca^2+^ transient peak requires further investigation. Fibroblasts have been proposed to increase hiPSC-CM sarcoplasmic reticulum Ca^2+^ content [47], however if fibroblasts affect ryanodine receptor-2 Ca^2+^ cycling kinetics remains unknown. It should be mentioned here that although hiPSC-CMs are the major contributors to optically recorded AP and Ca^2+^ transients in direct contact cocultures, fluorescence from MyoFBs could influence the overall signal. MyoFBs are nonexitable cells (no signals were detected in MyoFBs only cultures), however when electrotonically coupled to cardiomyocytes they could display AP-like signals [26].

Direct heterocellular contact increased hiPSC-CM spontaneous activity in our study similarly to previous work [34, 48, 49], an effect reported to be a result of heterocellular coupling independent from media conditioning [50]. Importantly, increased spontaneous activity of transplanted cells has been linked to arrhythmia in large animal studies [8].

MyoFBs reduced the ability of hiPSC-CMs to respond to increasing pacing frequencies in all culture conditions, resulting in loss of 1:1 AP capture and arrhythmic behaviour. APD prolongation and therefore longer refractory period are likely the mechanism leading to the loss of 1:1 capture in our study. Impaired ability of hiPSC-CMs to respond to the next stimulus could promote conduction block and the formation of a substrate for re-entrant arrhythmia [51]. hiPSC-CM APD prolongation would also increase the difference in APD between transplanted and resident cells, creating a substrate for arrhythmogenic activity [10].

Our results show that IL-6 plays a major role in MyoFBs–hiPSC-CMs paracrine communication. IL-6, a pro-inflammatory cytokine produced by both cardiomyocytes and fibroblasts [23, 52], is increased in post-MI patients [22] and elevated in patients with heart failure [24], where it correlates with increased risk of cardiovascular events and ventricular dysfunction [24, 53]. Blocking IL-6 with a neutralising antibody prevented/reduced the effect of MyoFBs on hiPSC-CM APD, Ca^2+^ decay and loss of 1:1 capture in noncontact coculture and in the presence of conditioned medium. However, in contact cocultures blocking IL-6 had no effect, suggesting that contact-mediated communication may be the predominant mechanism in these settings. Adding exogenous IL-6 directly to hiPSC-CMs recapitulated the prolongation of APD50/90 and Ca^2+^ decay observed in noncontact coculture and conditioned medium, supporting the conclusion that these effects are due to direct IL-6 signalling. IL-6 has been shown to prolong APD50 and APD90 in adult guinea-pig cardiomyocytes [54] and Ca^2+^ decay in adult mouse cardiomyocytes [55] through reduced *KCNH2* and *SERCA2a* mRNA expression respectively [56]. IL-6 involvement in *KCNH2* and *SERCA2a* mRNA downregulation in hiPSC-CMs warrants further investigation.

To dissect the mechanism underlying the effect of MyoFBs on hiPSC-CM function in contact cocultures, we knocked down Cx43 specifically in MyoFBs prior to culture in contact with hiPSC-CMs. Silencing of Cx43 expression in MyoFBs has previously been shown to be anti-arrhythmic, leading to reduced APD prolongation, ectopic activity and cell excitability in neonatal rat cardiomyocytes [34], whilst knockdown of Cx43 in non-cardiomyocytes cultured with hiPSC-CMs has been shown to increase upstroke velocity and reduce AP triangulation [57]. In contrast, we found that MyoFB-specific Cx43-KD did not prevent or reduce changes to hiPSC-CM electrophysiology. Instead, Cx43-KD appeared to promote a paracrine-mediated mechanism and a profile of functional changes similar to those observed in noncontact cocultures. This idea was further supported as IL-6 blockade in Cx43-KD contact cocultures was able to prevent/reduce changes to APD and Ca^2+^ decay. This suggests a dynamic interaction between gap junctional and paracrine signalling in contact cocultures, with gap junctional signalling dominating in the presence of Cx43-based heterocellular coupling while paracrine signalling dominates in the absence of Cx43-mediated interactions. Both uncoupling of MyoFBs and hiPSC-CMs and blockade of paracrine signalling appear to be needed to reduce electrophysiology and Ca^2+^ handling changes in this scenario, although further contribution of mechanical interactions or ephaptic coupling cannot be excluded [58, 59]. Crosstalk between gap junctional and paracrine signalling remains largely unexplored in the heart, however a study by Salvarani et al. demonstrated that both TGF-β1 signalling and MyoFB-cardiomyocyte coupling contribute to the changes in conduction and ectopic activity of neonatal rat cardiomyocytes in fibrotic coculture [60].

Cell-based therapy can improve cardiac function but has been associated with increased incidence of ventricular arrhythmias [3, 7, 9, 61, 62]. Focal activity, primarily enhanced automaticity, as well as re-entrant circuits have been proposed as mechanisms of arrhythmogenicity. Whilst the immature nature of transplanted grafts is an important factor, the influence of the fibrotic niche on graft electrical activity needs consideration [7, 63]. Fibroblasts have been shown to exert a negative influence on hiPSC-CMs maturation through both Cx43-mediated cell–cell contacts [57] and paracrine signalling [64, 65]. Electrophysiological heterogeneity at the site of hiPSC-CMs grafts may create a substrate for arrhythmogenic activity [10, 66] which can be further exacerbated by the MyoFB induced changes to APD or Ca^2+^ decay reported here. Our study identifies druggable pathways that could reduce the risks of cell therapy mediated arrhythmia. IL-6 is a potential therapeutic target to reduce APD and Ca^2+^ transient changes and decrease susceptibility of hiPSC-CMs to loss of capture and potential conduction block when cocultured with MyoFBs, which in-turn may lead to reduced electrophysiological heterogeneity at the site of hiPSC-CMs grafts and reduced risk of arrhythmia post-engraftment. The ongoing ASSAIL-MI trial has shown the potential for anti-IL-6 treatment to increase myocardial salvage in STEMI patients at the time of reperfusion [67], whilst previous evidence has highlighted the anti-arrhythmic potential of anti-IL-6R therapy in patients with rheumatoid arthritis and COVID-19 [53, 68]. Furthermore, given the number of connexin-targeted agents currently in clinical trials, which includes Cx43 antisense oligonucleotide and mimetic peptides [69], combining IL-6 therapy with connexin therapeutics could become a feasible avenue to improve the outcome of cardiac cell therapy.

Our study focused on the interaction between MyoFBs and hiPSC-CM, however MyoFBs present at the border zone are likely to also affect resident adult cardiomyocytes post-MI. *Ex-vivo* studies in Langendorff-perfused mouse hearts have shown that MyoFBs can electrically couple to cardiomyocytes in the infarct border zone [26–28, 70]. This coupling has recently been associated with proarrhythmic remodelling at the infarct border zone in aged rabbits [71]. In vitro studies indicate that paracrine communication could also promote adult cardiomyocyte remodelling [16, 20], however if this occurs in cultures of human cells or in the whole organ remains unknown. Further research investigating mechanisms of crosstalk between human MyoFBs and adult cardiomyocytes both in vitro and in situ is needed. The recent development of protocols to induce maturation of hiPSC-CM will benefit future in vitro work [72].

In our study we used iCell hiPSC-CMs (Cellular Dynamics International) and it is possible that alternative cell lines may respond differently. The experimental protocols presented here could be used to identify hiPSC-CMs with the most favourable properties for grafting. This would be particularly valuable for screening the clinical-grade haplobanks which are being generated to provide iPSCs matched to human leukocyte antigens to avoid immune-rejection [73].

In conclusion, this study shows that human cardiac MyoFBs alter both electrical and Ca^2+^ handling properties of hiPSC-CMs through distinct paracrine-mediated and contact-mediated mechanisms, leading to electrophysiological changes which promote loss of 1:1 AP capture, potential conduction block and increased susceptibility to arrhythmia. This study also highlights the complex interplay of paracrine-signalling and heterocellular coupling when MyoFBs and hiPSC-CMs are in contact. The pivotal role of IL-6 and Cx43-signalling identified here highlights these pathways as potential therapeutic targets to reduce the risk of arrhythmia following hiPSC-CMs transplant post-MI.

## Supplementary Information

Below is the link to the electronic supplementary material.

Supplementary file1 (DOCX 1784 kb)

## Data Availability

The raw data supporting the conclusions of this manuscript are included in this manuscript and Supplementary Information.
