# Correction: Human myofibroblasts increase the arrhythmogenic potential of human induced pluripotent stem cell-derived cardiomyocytes

**DOI:** 10.1007/s00018-024-05492-w

**Published:** 2024-12-27

**Authors:** Robert D. Johnson, Ming Lei, John H. McVey, Patrizia Camelliti

**Affiliations:** 1 School of Biosciences, University of Surrey, Guildford, UK; 2 Department of Pharmacology, University of Oxford, Oxford, UK

**Correction: Cellular and Molecular Life Sciences (2023) 80:276**

10.1007/s00018-023-04924-3

In this article the below mentioned keywords was missed in the article.

- Cardiac Fibroblast-Cardiomyocyte Interactions
- Crosstalk between cardiac fibroblasts and myocytes

The original article has been corrected.

