# Supplementary material for: Human myofibroblasts increase the arrhythmogenic potential of human induced pluripotent stem cell-derived cardiomyocytes
Source: Cell Mol Life Sci. 2023 Sep 5;80(9):276. doi: 10.1007/s00018-023-04924-3 (PMC10480244; doi:10.1007/s00018-023-04924-3)
Supplement: Supplementary file 1 — Supplementary file1 (DOCX 1784 kb) [file 18_2023_4924_MOESM1_ESM.docx]

**Supplementary information**


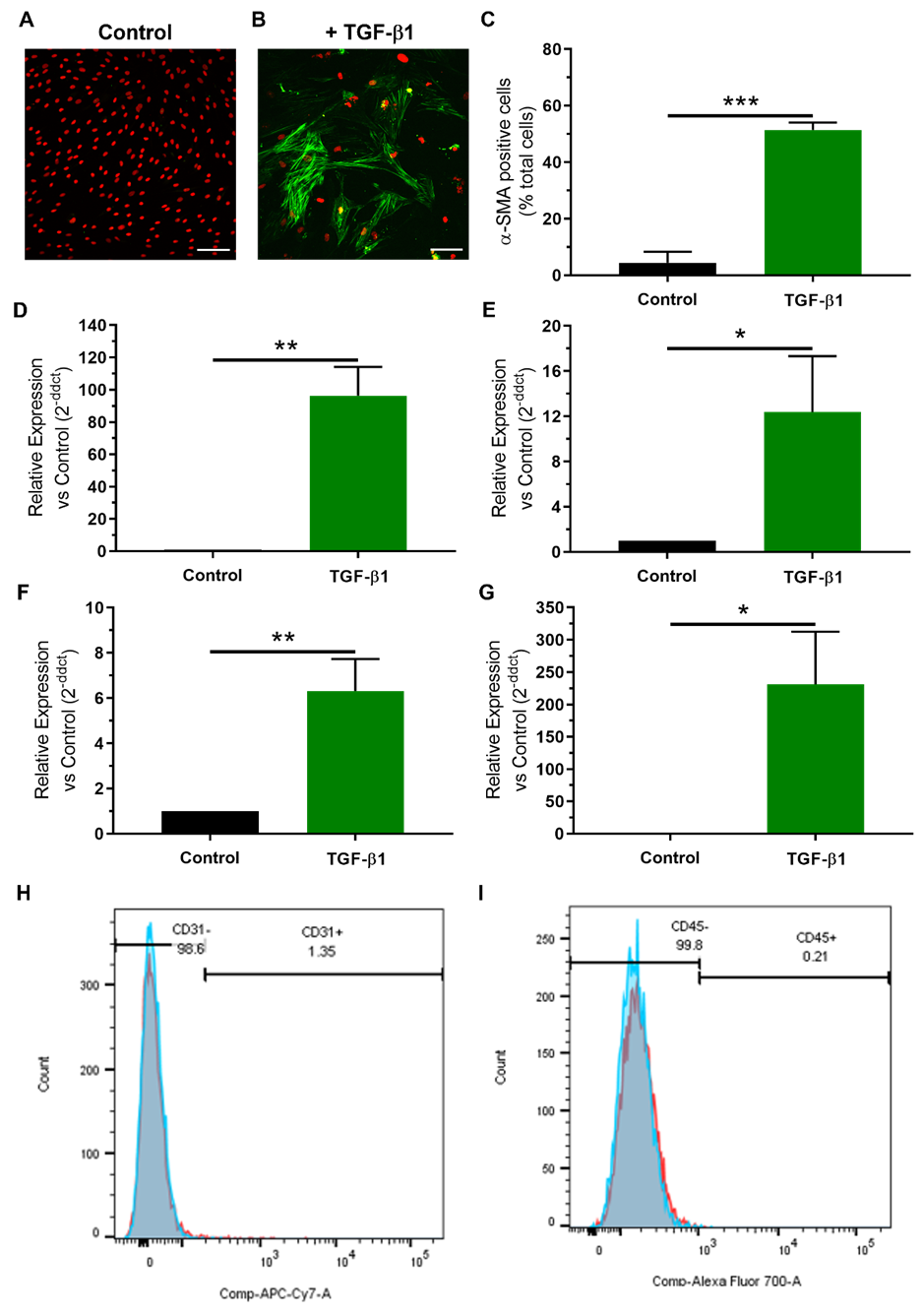


**Supplementary Fig. 1: Characterisation of adult human myofibroblasts obtained from TGFβ1-treated primary human cardiac fibroblast cultures.**

**A, B** Representative confocal images showing α-SMA (green) staining prior (**A**, control) and post activation (**B**, TGF-β1 5 ng/mL, 48 h) of primary fibroblasts into myofibroblasts. Propidium iodide (red) marks nuclei. Scale bar: 100 µm. **C** Quantification of α-SMA positive cells as a percentage of all cells (n = 3 independent experiments per group). **D-G** Quantification by RT-qPCR of α-SMA (ACTA2) **(D),** Col1A1 **(E)**, IL-6 **(F)**, and IL-11 **(G)** following TGF-β1 treatment (n = 3 independent experiments per group). Values expressed as means ± SEM. *p<0.05, **p<0.01, ***p<0.001 (Student’s unpaired t-test). **H, I** Histogram plot of flow cytometry analysis for CD31 (**H**) and CD45 (**I**) in TGF-β1 activated cultures. Blue lines in the histogram are the isotype controls. Red lines show the particular marker. All cell samples used in the measurements are from passages 3 to 5.


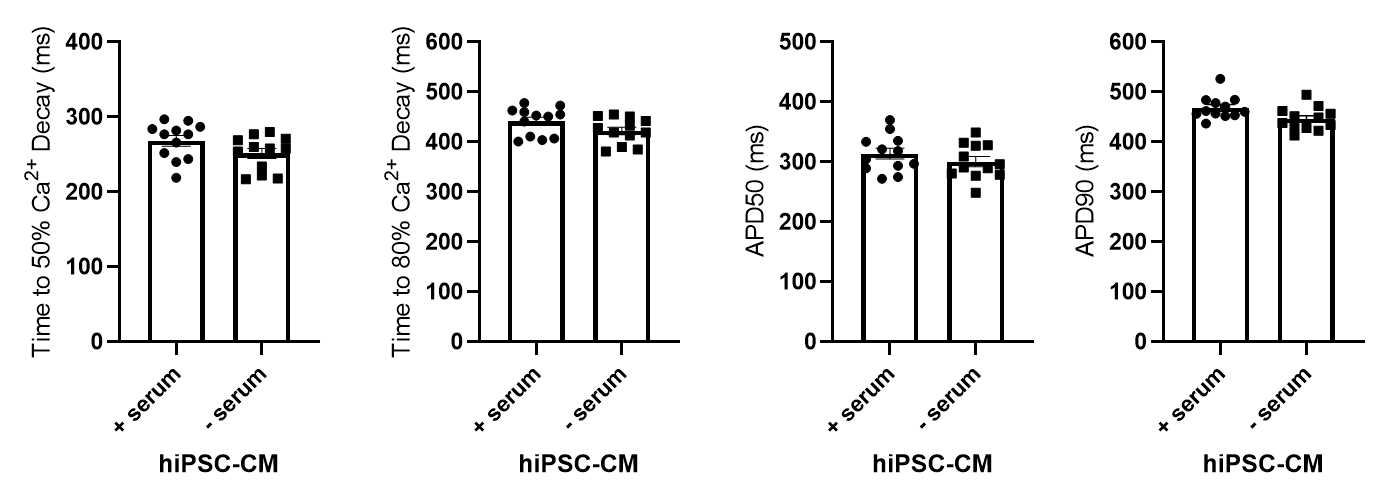


**Supplementary Fig. 2: Characterisation of hiPSC-CM monolayers in the presence and absence of serum.** hiPSC-CMs were cultured for 48 h in serum containing DMEM or serum-free DMEM prior to optical mapping characterisation. No differences were observed in Ca^2+^ transients (time to 50% Ca^2+^ decay and time to 80% Ca^2+^ decay) and action potential parameters (APD50 and APD90). Bars represent mean ± SEM (n = 12 monolayers). p>0.5 (unpaired t-test).

**
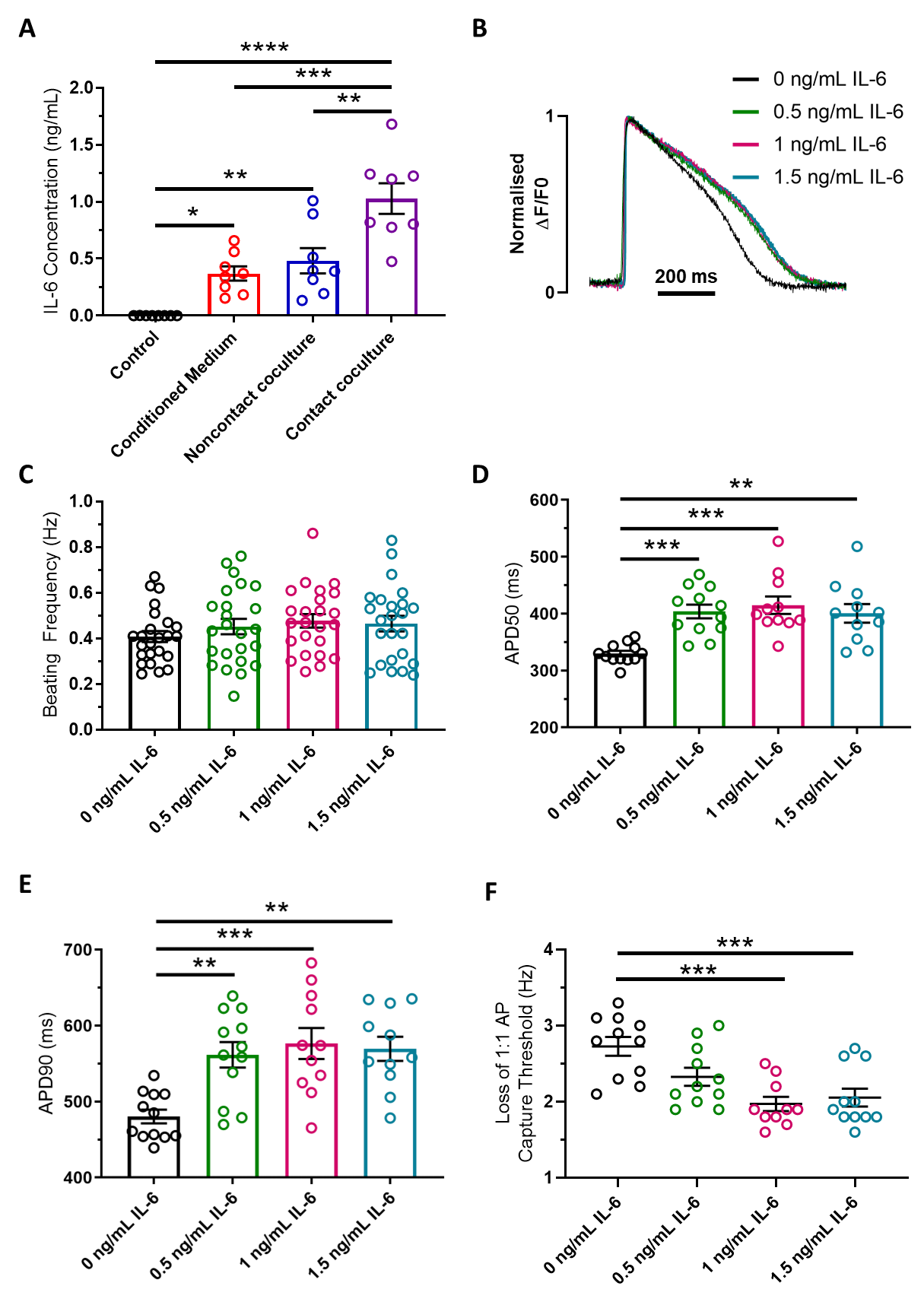
**

**Supplementary Fig. 3: Effect of IL-6 stimulation on hiPSC-CM electrical function.**

**A** Average IL-6 concentration in media samples from hiPSC-CMs cultured with MyoFBs in the three conditions: with MyoFBs conditioned medium (red), noncontact (blue) and contact coculture (purple), versus control (black). Values expressed as means ± SEM (n = 8 media samples). **B** Superimposed representative optical action potentials from hiPSC-CMs stimulated with 0.5 ng/mL (green), 1 ng/mL (pink) and 1.5 ng/mL (turquoise) IL-6. Control (black): hiPSC-CMs cultured with basal DMEM media. Cells were electrically stimulated at 1 Hz. **C** Average spontaneous beating frequency of hiPSC-CM stimulated with 0 ng/mL, 0.5 ng/mL, 1 ng/mL and 1.5 ng/mL IL-6. Values expressed as means ± SEM (n = 24 treated monolayers). **D-E** Summary of APD50 (**D**) and APD90 (**E**) changes for hiPSC-CMs stimulated with the different concentrations of IL-6. Values expressed as means ± SEM (n = 11-12 treated monolayers). **F** Average pacing rate needed to induce loss of 1:1 action potential capture in hiPSC-CM cultures following IL-6 treatment. Values expressed as means ± SEM (n = 10-11 treated monolayers). *p<0.05, **p<0.01, ***p< 0.001, ****p< 0.0001 (one‐way ANOVA with post hoc Tukey's test).


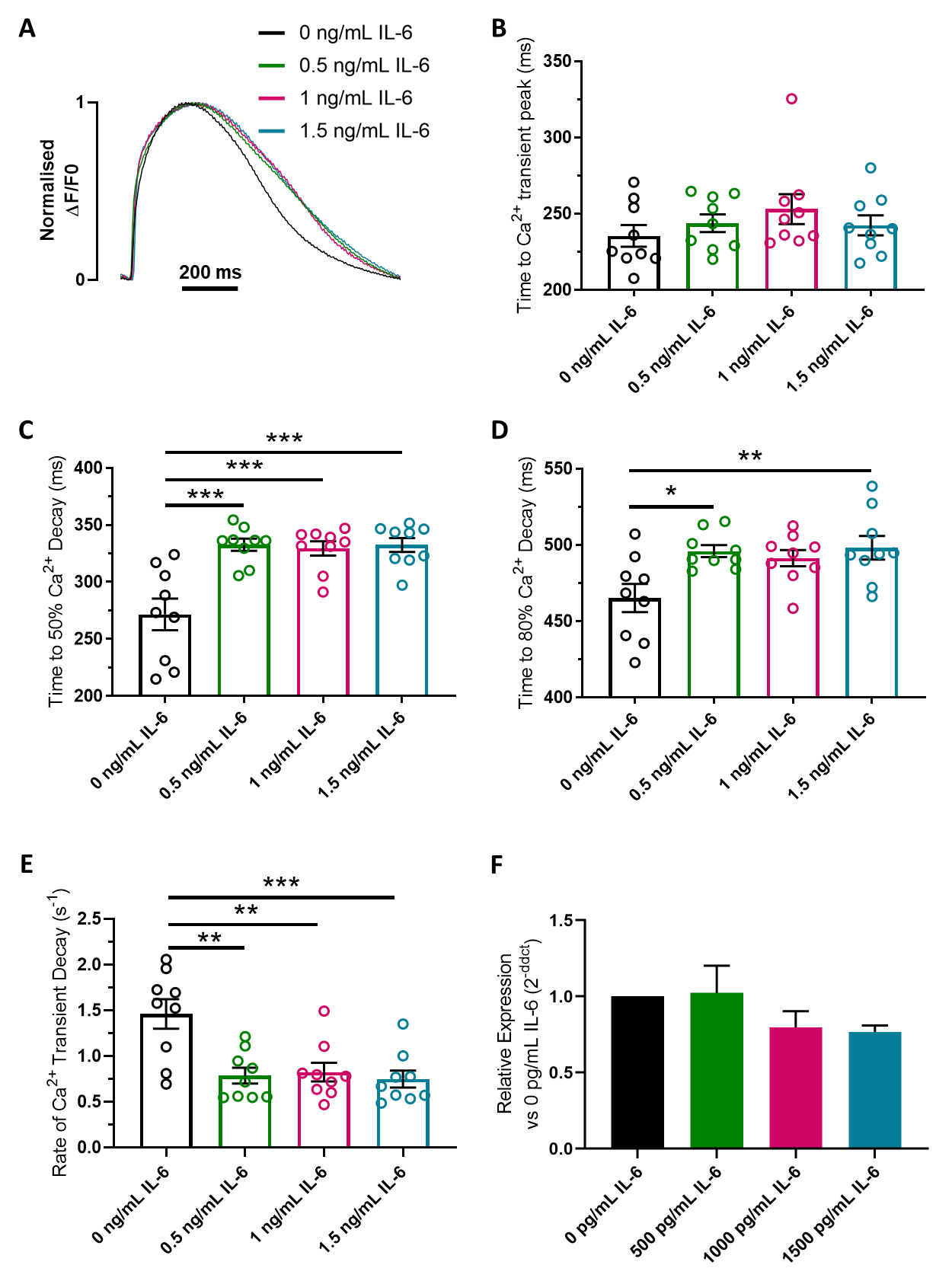


**Supplementary Fig. 4: Effect of IL-6 stimulation on hiPSC-CM calcium handling and interleukin-6 receptor mRNA.**

**A** Superimposed representative optical Ca^2+^ transients from hiPSC-CMs cultured in the presence of 0.5 ng/mL (green), 1 ng/mL (pink) and 1.5 ng/mL (turquoise) IL-6. Control (black): hiPSC-CM cultured with basal DMEM media. Cells were electrically stimulated at 1 Hz. **B-E** Time to Ca^2+^ transient peak (**B**), time to 50% Ca^2+^ decay (**C**), time to 80% Ca^2+^ decay (**D**) and rate of Ca^2+^ transient decay (**E**) for hiPSC-CMs cultured in the absence or presence of IL-6 at 3 different concentrations. Values expressed as means ± SEM (n = 9 treated monolayers). *p<0.05, **p<0.01, ***p<0.001 (one‐way ANOVA with post hoc Tukey's test). **F** Changes in the abundance of mRNA encoding the interleukin-6 receptor (IL-6R) in hiPSC-CMs stimulated with 0, 0.5, 1 and 1.5 ng/mL IL-6. Values expressed as means ± SEM (n = 3 independent cultures). p>0.05 (one‐way ANOVA with post hoc Dunnet’s test versus 0 ng/mL IL-6).

**
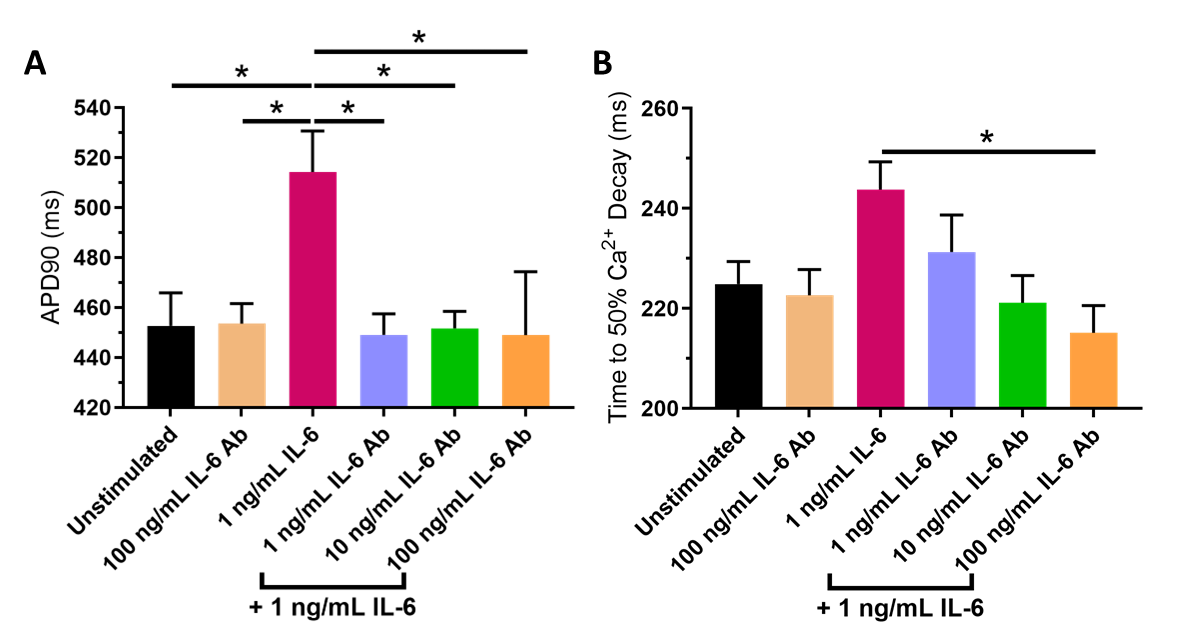
**

**Supplementary Fig. 5: Neutralising IL-6 antibody prevents IL-6-stimulated APD and calcium decay prolongation.**

hiPSC-CMs were either unstimulated or treated with 100 ng/mL IL-6 neutralising antibody (IL-6 Ab) alone, 1 ng/mL IL-6 alone, 1 ng/mL IL-6 Ab + 1 ng/mL IL-6, 10 ng/mL IL-6 Ab + 1 ng/mL IL-6, or 100 ng/mL IL-6 Ab + 1 ng/mL IL-6. **A** Summary of APD90 changes in hiPSC-CMs from the different IL-6 stimulation conditions, at 1 Hz pacing rate. Values expressed as means ± SEM (n = 4 treated monolayers). **B** Summary of time to 50% Ca^2+^ decay changes in hiPSC-CMs from the different IL-6 stimulation conditions, at 1 Hz pacing rate. Values expressed as means ± SEM (n = 3-4 treated monolayers). *p<0.05 (one‐way ANOVA with post hoc Dunnet’s test versus 1 ng/mL IL-6 alone).

**
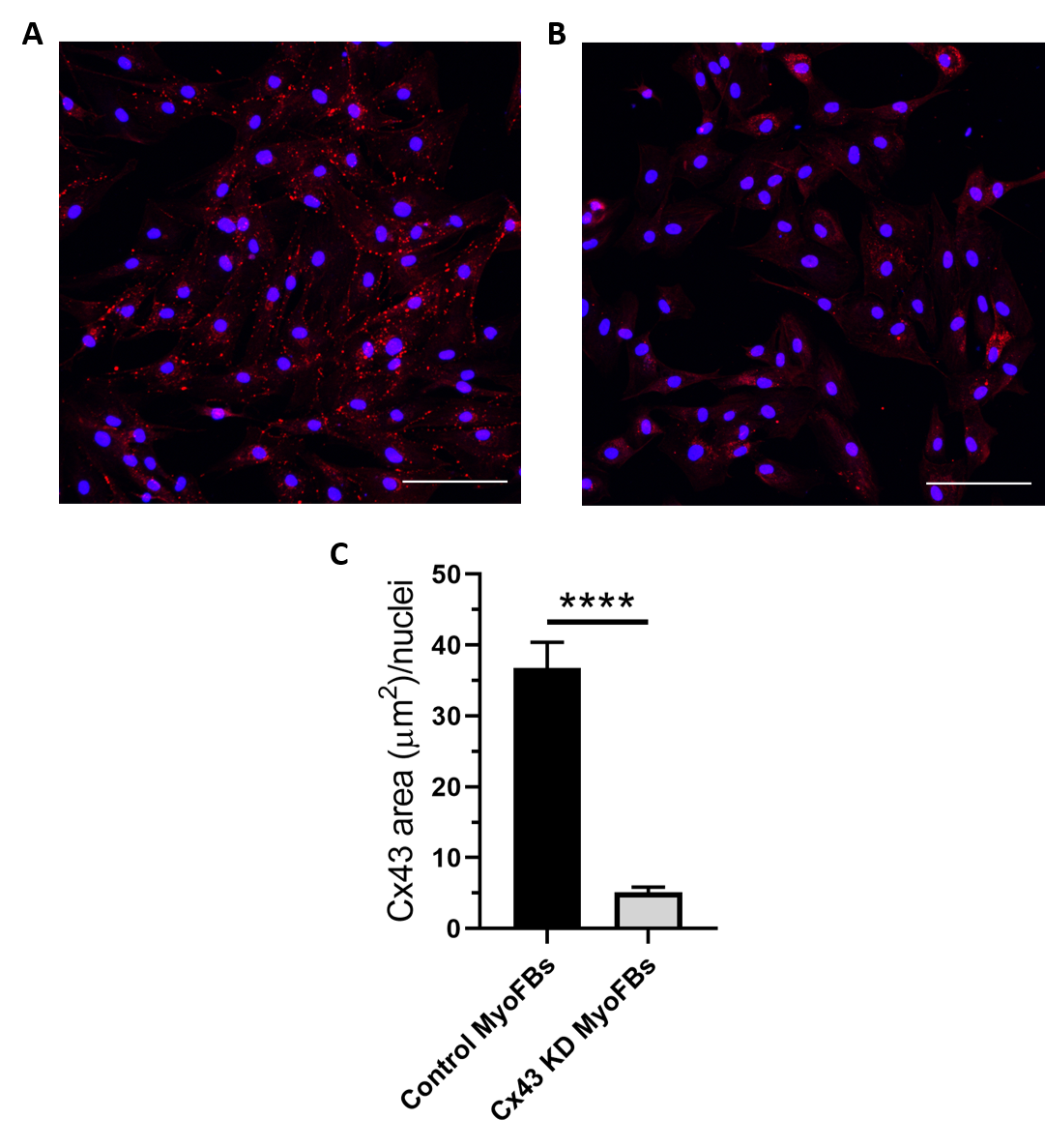
**

**Supplementary Fig. 6: Validation of Cx43 protein knockdown in myofibroblasts.**

**A,B** Representative confocal fluorescent images of cardiac myofibroblasts stained for Cx43 (red) under control conditions (**A**) or following transfection for 24 h with Cx43 siRNA 2 (**B**). Hoechst-33342 (blue) marks nuclei. Scale bar: 100 µm. **C** Quantification of Cx43 expression in control and Cx43-KD MyoFBs as a function of mean fluorescent area/number of nuclei ± SEM (control: n = 17 areas, 1240 cells; Cx43-KD: n = 19 areas, 1068 cells). ****p<0.0001 (Student’s unpaired t-test).


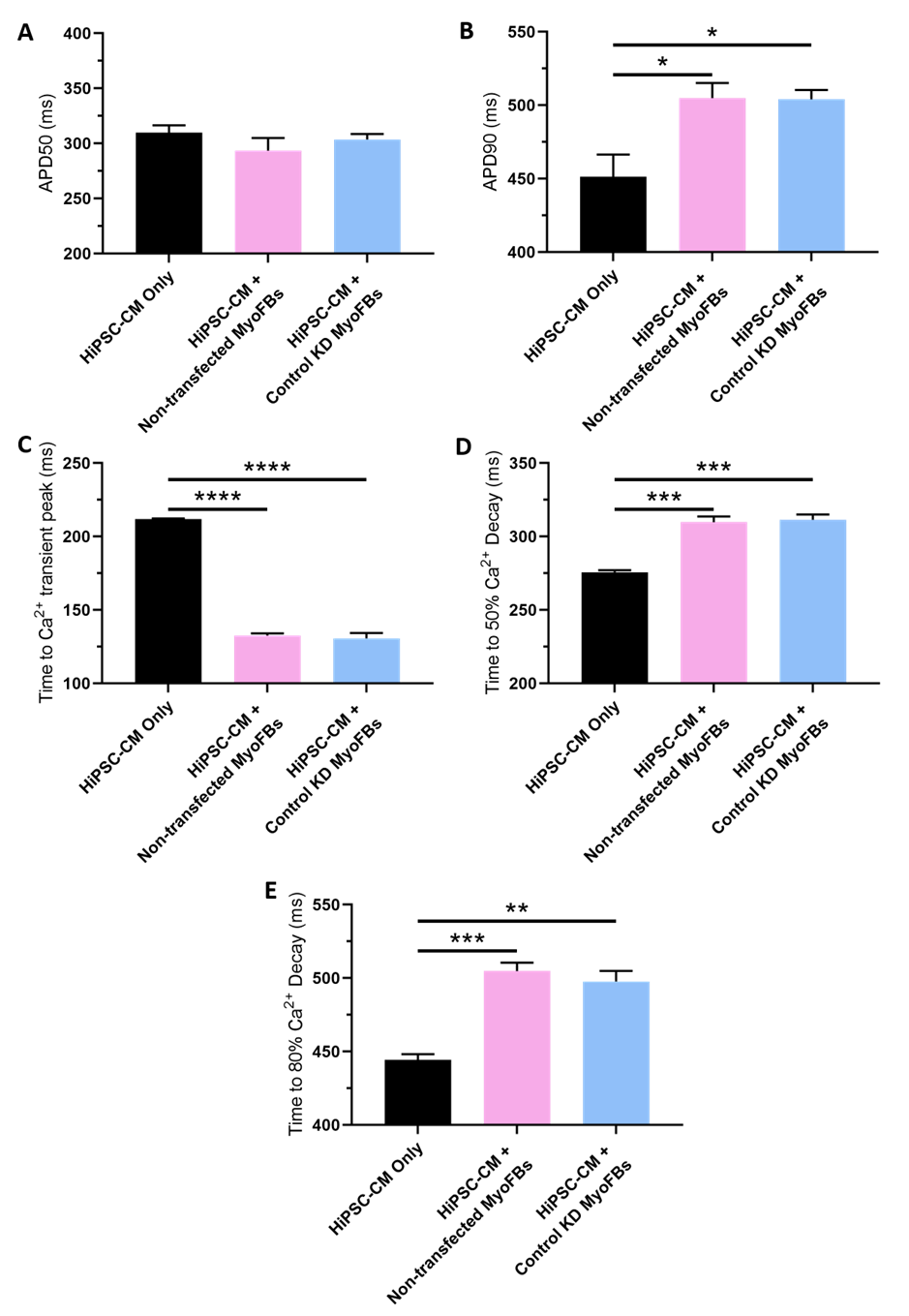


**Supplementary Fig. 7: Comparison of hiPSC-CM electrical function and calcium handling following contact coculture with non-transfected myofibroblasts and myofibroblasts transfected with a negative control siRNA.**

**A-E** Summary of APD50 (**A**), APD90 (**B**), time to Ca^2+^ transient peak (**C**), time to 50% Ca^2+^ decay (**D**), and time to 80% Ca^2+^ decay (**E**) for hiPSC-CM cultured alone (HiPSC-CM only), in contact with non-transfected MyoFBs (HiPSC-CM + Non-transfected MyoFBs) or in contact with MyoFBs transfected with the negative control siRNA (HiPSC-CM + Control KD MyoFBs) at 1 Hz pacing rate. Values expressed as means ± SEM (n = 3 treated monolayers). *p<0.05, **p<0.01, ***p<0.001, ****p<0.0001 (one‐way ANOVA with post hoc Tukey's test).

**Supplementary Table 1: Primers for RT-qPCR**

| **Gene name** | **Applied Biosystems Taqman Assay ID** |
| --- | --- |
| B2M | Hs00984230_m1 |
| ACTA2 | Hs00426835_g1 |
| Col1A1 | Hs00164004_m1 |
| IL-6 | Hs00174131_m1 |
| IL-11 | Hs01055413_g1 |
| CD90 | Hs00174816_m1 |
| CD31 | Hs01065279_m1 |
| CD45 | Hs04189704_m1 |
| ATP2A2 | Hs01564005_m1 |
| SLC8A1 | Hs01062258_m1 |
| CACNA1C | Hs00167681_m1 |
| KCNH2 | Hs04234270_g1 |
| KCNJ2 | Hs00265315_m1 |
| NPPB | Hs00173590_m1 |
| GJA1 | Hs00748445_s1 |
| GAPDH | Hs02786624_g1 |
